# Synthetic essentiality between PTEN and core dependency factor PAX7 dictates rhabdomyosarcoma identity

**DOI:** 10.1038/s41467-021-25829-4

**Published:** 2021-09-17

**Authors:** Casey G. Langdon, Katherine E. Gadek, Matthew R. Garcia, Myron K. Evans, Kristin B. Reed, Madeline Bush, Jason A. Hanna, Catherine J. Drummond, Matthew C. Maguire, Patrick J. Leavey, David Finkelstein, Hongjian Jin, Patrick A. Schreiner, Jerold E. Rehg, Mark E. Hatley

**Affiliations:** 1 Department of Oncology, St. Jude Children’s Research Hospital, Memphis, TN 38105 USA; 2 Rhodes College, Memphis, TN 38112 USA; 3 St. Jude Graduate School of Biomedical Sciences, Memphis, TN 38105 USA; 4 Department of Computational Biology, St. Jude Children’s Research Hospital, Memphis, TN 38105 USA; 5 Center for Applied Bioinformatics, St. Jude Children’s Research Hospital, Memphis, TN 38105 USA; 6 Department of Pathology, St. Jude Children’s Research Hospital, Memphis, TN 38105 USA; 7 Present Address: Purdue Center for Cancer Research, Department of Biological Sciences, Purdue University, West Lafayette, IN USA; 8 Present Address: Department of Pathology, University of Otago, Dunedin, Otago New Zealand

**Keywords:** Cancer models, Embryonal neoplasms, Paediatric cancer, Sarcoma, Tumour-suppressor proteins

## Abstract

*PTEN* promoter hypermethylation is nearly universal and *PTEN* copy number loss occurs in ~25% of fusion-negative rhabdomyosarcoma (FN-RMS). Here we show *Pten* deletion in a mouse model of FN-RMS results in less differentiated tumors more closely resembling human embryonal RMS. PTEN loss activated the PI3K pathway but did not increase mTOR activity. In wild-type tumors, PTEN was expressed in the nucleus suggesting loss of nuclear PTEN functions could account for these phenotypes. *Pten* deleted tumors had increased expression of transcription factors important in neural and skeletal muscle development including *Dbx1* and *Pax7*. *Pax7* deletion completely rescued the effects of *Pten* loss. Strikingly, these *Pten;Pax7* deleted tumors were no longer FN-RMS but displayed smooth muscle differentiation similar to leiomyosarcoma. These data highlight how *Pten* loss in FN-RMS is connected to a PAX7 lineage-specific transcriptional output that creates a dependency or synthetic essentiality on the transcription factor PAX7 to maintain tumor identity.

## Abstract (web summary)

PTEN copy number loss is found in 25% of fusion-negative rhabdomyosarcomas (FN-RMS). Here, the authors use a Hedgehog-driven FN-RMS mouse model to show that PTEN loss drives the expression of core transcription factor PAX7 and its transcriptional axis, which determines FN-RMS tumour identity.

## Introduction

Rhabdomyosarcoma (RMS) is the most common soft-tissue sarcoma in childhood and high-risk RMS patients have a 5-year survival rate of only 20%^1^. RMS is classified into two major histologic subgroups, alveolar (ARMS) and embryonal (ERMS)^2^. The majority of ARMS tumors (~80%) harbor chromosomal translocations resulting in expression of either PAX3-FOXO1 or PAX7-FOXO1 oncofusion proteins which foretell a worse prognosis^1^. ARMS tumors without oncofusion proteins both molecularly and clinically resemble ERMS, thus classification as either fusion-positive (FP-RMS) or fusion-negative (FN-RMS) better reflects the biological and clinical features of RMS^1^. FN-RMS tumors are genetically heterogeneous with a constellation of putative driver mutations and loss-of-function alterations in tumor suppressors such as *TP53* and *CDKN2A*; the majority of tumors have no apparent driver mutations^3–7^. In contrast, hypermethylation of the Phosphatase and Tensin Homolog (*PTEN*) promoter and decreased expression was found in over 90% of FN-RMS tumors^6^. *PTEN* promoter hypermethylation is one of many ways that *PTEN* expression can be modulated in cancer^8,9^. Furthermore, a subset of FN-RMS tumors also harbors *PTEN* mutations^10^.

PTEN’s tumor-suppressive function is thought to be secondary to dephosphorylation and inactivation of the lipid second messenger phosphatidylinositol-3,4,5-triphosphosphate, a potent PI3K/AKT/mTOR pathway activator^11–13^. Inhibition of the PI3K/AKT/mTOR pathway is an attractive RMS therapy but has yielded varied results^3,14–16^. However, increasing evidence suggests PTEN maintains a myriad of tumor-suppressive functions aside from negatively regulating the PI3K pathway^11^. *PTEN-*deficient tumors require the expression of other genes to maintain their proliferative state; these synthetic essential gene interactions create therapeutically exploitable gene dependencies^17,18^. Using an integrative expression and copy number computational approach, PTEN was suggested to be a key tumor suppressor in FN-RMS^19^. However, PTEN’s biological function in FN-RMS is largely unexplored. Herein, we leverage a Hedgehog-driven, FN-RMS mouse model to interrogate the role of PTEN in FN-RMS biology^20,21^. In contrast to modulating the PI3K/AKT/mTOR pathway, we reveal PTEN loss drives a transcriptional axis centered on PAX7 necessary for FN-RMS tumor identity.

## Results

### *Pten* loss exacerbates *aP2-Cre;SmoM2* FN-RMS development

The nearly universal *PTEN* promoter hypermethylation in FN-RMS^6^ led us to investigate whether *Pten* loss is sufficient to drive rhabdomyosarcomagenesis by conditionally deleting a *Pten*^*flox*^ allele with Cre recombinase expressed from the *adipose protein 2* (aP2) promoter (Supplementary Fig. 1a)^22^. Neither *aP2-Cre;Pten*^*flox/flox*^ (AP^cKO^) nor *aP2-Cre;Pten*^*flox/+*^ (AP^cHet^) compound mutant mice developed tumors. AP^cKO^ mice had significantly larger intrascapular brown adipose tissue (BAT) but not inguinal white adipose tissue (iWAT) than AP^cHet^ littermates (Supplementary Fig. 1b–d). Histologically, the BAT was hyperplastic without evidence of hibernoma (Supplementary Fig. 1e). The iWAT displayed hyperplastic cellularity without any overt pathology (Supplementary Fig. 1f). Thus, *Pten* loss is not sufficient to drive rhabdomyosarcomagenesis in *aP2-Cre* mice.

**Fig. 1:**
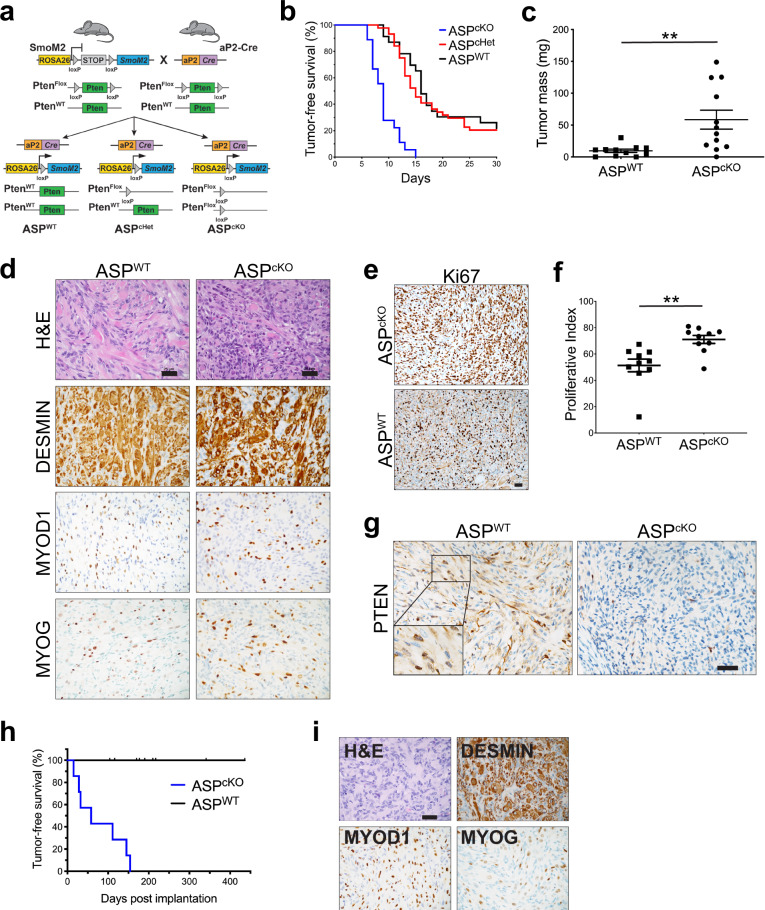
*Pten* loss exacerbates *aP2-Cre;SmoM2* FN-RMS development. **a** Breeding strategy to generate ASP^WT^, ASP^cHet^, and ASP^cKO^ mice. **b** Kaplan–Meier tumor-free survival in ASP^WT^ (*n* = 23), ASP^cHet^ (*n* = 42), and ASP^cKO^ (*n* = 18) tumors (*P* < 0.0001). **c** Tumor mass at P12 from ASP^WT^ (*n* = 11) and ASP^cKO^ (*n* = 12) mice (*P* = 0.0052). **d** Representative histology of ASP^WT^ (*n* = 16) and ASP^cKO^ (*n* = 16) tumors by H&E and IHC staining for DESMIN, MYOD1, and MYOGENIN (MYOG). Scale bar, 25 μm. **e**, **f** Representative Ki67 IHC (**e**) and quantitation (**f**) in ASP^WT^ (*n* = 9) and ASP^cKO^ tumors (*n* = 7) tumors (ten random fields of view per genotype) (*P* = 0.0029). Scale bar, 25 μm. **g** Representative PTEN IHC in ASP^WT^ (*n* = 3) and ASP^cKO^ (*n* = 3) tumors. Scale bar, 25 μm. **h** Kaplan–Meier tumor-free survival curve of subcutaneous flank allografts of ASP^WT^ (*n* = 11) and ASP^cKO^ (*n* = 7) tumors (*P* < 0.0001). **i** Representative histology of ASP^cKO^ (*n* = 3) allografts by H&E staining and IHC for DESMIN, MYOD1, and MYOG. Scale bar, 25 μm. All pairwise comparisons determined by unpaired, two-tailed Student’s *t* test. Kaplan–Meier survival curves analyzed with Mantel–Cox log-rank test. **P < 0.01, mean ± SEM. See also Supplementary Figs. 1 and 2. Source data are provided as a Source Data file.

In order to understand the consequences of PTEN loss in FN-RMS, we sought to conditionally delete *Pten*^*flox*^ in our murine FN-RMS model resulting from activation of a conditional, constitutively active Smoothened allele (SmoM2) by aP2-Cre^20,21^. We bred *aP2-Cre;Pten*^*flox/+*^ mice to *Smo*^*M2/M2*^*;Pten*^*flox/+*^ mice to generate *aP2-Cre;Smo*^*M2/+*^*;Pten*^*flox/flox*^ *(*ASP^cKO^), *aP2-Cre;Smo*^*M2/+*^*;Pten*^*flox/+*^ (ASP^cHet^), and *aP2-Cre;Smo*^*M2/+*^*;Pten*^*+/+*^ (ASP^WT^) compound mutant mice (Fig. 1a). *Pten* loss accelerated tumor formation with a median tumor-free survival of 9 days in ASP^cKO^ mice compared to 16 days in ASP^WT^ mice (Fig. 1b and Supplementary Fig. 2a). All tumors in ASP^cKO^ mice occurred in the head and neck without any evidence of regional lymph node or pulmonary metastasis at necropsy just as in the ASP^WT^ mice. In addition, ASP^cKO^ mice had increased tumor burden and decreased body weight compared to ASP^WT^ mice (Fig. 1c and Supplementary Fig. 2b–d).

Histologic examination revealed ASP^cKO^ tumors contained malignant proliferations with vague, haphazard spindle cell growth patterns comprised of cohesive cells with coalescing pale pink cytoplasm, with or without striations and irregular margins infiltrating adjacent tissue (Fig. 1d). The cells contained variably sized round, oval, or spindle nuclei. By immunohistochemistry (IHC), ASP^cKO^ tumors strongly expressed muscle-specific intermediate filament DESMIN and nuclear staining for skeletal muscle-specific transcription factors MYOD1 and MYOGENIN (Fig. 1d and Supplementary Fig. 2e, f). In addition, ASP^cKO^ tumors have a higher proliferative index than ASP^WT^ tumors (Fig. 1e, f). The infiltrative pattern and cytological morphology of the small cells in ASP^cKO^ tumors are consistent with an embryonal-like RMS rather than the well-differentiated RMS more typical of the ASP^WT^ tumors. Importantly, PTEN protein expression by IHC showed the absence of PTEN staining in ASP^cKO^ tumors, whereas both cytoplasmic and nuclear PTEN staining was observed in ASP^WT^ tumors (Fig. 1g). These histologic observations suggest PTEN loss may have a functional role in maintaining a less differentiated cell state.

ASP^WT^ tumors can be serially transplanted in NSG but not in SCID/Beige immunocompromised mice (Supplementary Fig. 2g). To test whether tumors from ASP^cKO^ mice are more tumorigenic than ASP^WT^ mice, we surgically implanted tumors into the flanks of SCID/Beige mice. All ASP^cKO^ tumors implanted developed tumors that were histologically similar to FN-RMS suggesting ASP^cKO^ tumors are more malignant than ASP^WT^ tumors (Fig. 1h, i). The shorter latency, increased tumor burden, and enhanced tumorigenicity illustrates ASP^cKO^ tumors represent a more robust model of FN-RMS.

To determine if the effects of *Pten* loss were not solely reflective of generic tumor suppressor loss, we conditionally deleted *Trp53*, *Cdkn2a*, and *Rb1* independently in our *aP2-Cre;SmoM2* model. The deletion of *Trp53*, *Cdkn2a* or *Rb1* in our model did not alter the tumor latency, histology, or tumor burden despite increasing the penetrance of tumor formation (Supplementary Fig. 2h–q). While all ASP^cKO^ primary tumors formed secondary tumors as allografts in SCID/Beige mice, only two of six *aP2-Cre;Smo*^*M2/+*^*;Trp53*^*flox/flox*^ and two of eight *aP2-Cre;Smo*^*M2/+*^*;Rb1*^*flox/flox*^ formed secondary tumors while no tumors formed in *aP2-Cre;Smo*^*M2/+*^*;Cdkn2a*^*flox/flox*^ allografts (Supplementary Fig. 2q). These data suggest that *Pten* loss provides a significant advantage to the RMS tumor cell.

### PTEN loss enhances myogenic differentiation impairment in ASP^cKO^ tumors

**Fig. 2:**
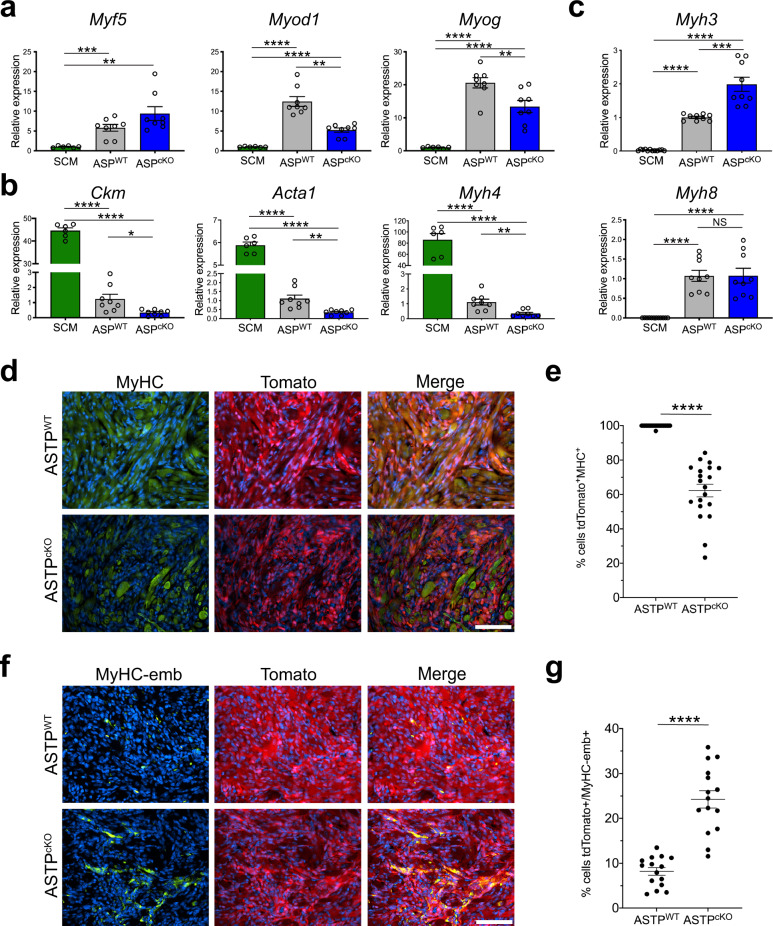
PTEN loss enhances myogenic differentiation impairment in ASP^cKO^ tumors. **a** Gene expression by real-time PCR of *Myf5*, *Myog*, and *Myod1* in sternocleidomastoid (SCM) muscles (*n* = 3), ASP^WT^ (*n* = 4), and ASP^cKO^ (*n* = 4) tumors, in duplicate. (*Myf5* SCM-ASP^WT^ *P* = 0.004, SCM-ASP^cKO^ *P* = 0.0014) (*Myod1* ASP^WT^-ASP^cKO^ *P* = 0.0001, SCM-ASP^WT^ *P* < 0.0001, SCM-ASP^cKO^ *P* < 0.0001) (*Myog* ASP^WT^-ASP^cKO^ *P* = 0.0099, SCM-ASP^WT^ *P* < 0.0001, SCM-ASP^cKO^ *P* < 0.0001). **b** Gene expression by real-time PCR of *Acta1, Myh4*, and *Ckm* in SCM (*n* = 3), ASP^WT^ (*n* = 4), and ASP^cKO^ (*n* = 4) tumors, in duplicate. (*Ckm* ASP^WT^-ASP^cKO^ *P* = 0.0136, SCM-ASP^WT^ *P* < 0.0001, SCM-ASP^cKO^ *P* < 0.0001) (*Acta1* ASP^WT^-ASP^cKO^ *P* = 0.0024, SCM-ASP^WT^ *P* < 0.0001, SCM-ASP^cKO^ *P* < 0.0001) (*Myh4* ASP^WT^-ASP^cKO^ *P* = 0.0027, SCM-ASP^WT^ *P* < 0.0001, SCM-ASP^cKO^ *P* < 0.0001). **c** Gene expression by real-time PCR of *Myh3* and *Myh8*, myosin heavy chains. SCM (*n* = 3), ASP^WT^ (*n* = 4), and ASP^cKO^ (*n* = 4) tumors, in triplicate. (*Myh3* ASP^WT^-ASP^cKO^ *P* = 0.0003, SCM-ASP^WT^ *P* < 0.0001, SCM-ASP^cKO^ *P* < 0.0001) (*Myh8* SCM-ASP^WT^ *P* < 0.0001, SCM-ASP^cKO^ *P* < 0.0001. **d**, **e** Representative immunofluorescence (IF) from ASTP^WT^ and ASTP^cKO^ tumors stained for mature myosin heavy chain (MyHC, green) with endogenous tdTomato fluorescence (red). DAPI nuclear stain (blue). Representative images shown, *n* = 3 tumors per genotype. Scale bar, 100 μm. **e** Quantification of tdTomato + /MHC + cells in tumors from (**d**) (*P* < 0.0001). **f**, **g** Representative IF from ASTP^WT^ and ASTP^cKO^ tumors stained for embryonic myosin heavy chain (MyHC-emb, green) with endogenous tdTomato fluorescence (red). DAPI nuclear stain (blue). Representative images shown, *n* = 3 tumors per genotype. Scale bar, 100 μm. **g** Quantification of tdTomato + /MyHC-emb + cells in tumors from (**f**) (*P* < 0.0001). All pairwise comparisons were determined by Student’s *t* test (unpaired, two-tailed); *P < 0.05, **P < 0.01, ***P < 0.001, ****P < 0.0001. Data represented as mean + SEM. Real-time PCR normalized to 18S rRNA and expressed relative to SCM (**a**) or ASP^WT^ (**b**, **c**). See also Supplementary Fig. 3. Source data are provided as a Source Data file.

Skeletal muscle development is a well-studied process of tissue differentiation driven by transcriptional regulation from fetal to mature adult skeletal muscle tissue^23^. Both ASP^cKO^ and ASP^WT^ tumors had increased expression of early skeletal muscle transcription factors *Myod1*, *Myf5*, and *Myog* compared to the mature sternocleidomastoid (SCM) skeletal muscle by real-time PCR consistent with FN-RMS (Fig. 2a). Although there were differences in *Myod1* and *Myog* mRNA expression between ASP^WT^ and ASP^cKO^ tumors, there was no change in protein expression by IHC (Fig. 1d and Supplementary Fig. 2e, f). In contrast, ASP^cKO^ tumors had significantly lower expression of terminally differentiated skeletal muscle genes, *Acta1*, *Ckm*, and *Myh4* (Fig. 2b). This suggests that *Pten* loss further attenuates the differentiation of the skeletal myogenic program within the FN-RMS tumor cells.

Similar to the series of transcription factors driving skeletal muscle determination and differentiation, myosin heavy chain (MyHC) isoforms of sarcomeric filaments display a developmental progression from MyHC-embryonic (*MYH3*) to MyHC-neonatal (*MYH8*) to finally adult MyHCs (*MYH1*, *MYH2*, *MYH4*)^23^. MyHC-embryonic encoded by *Myh3* which is only expressed in embryonic developing skeletal muscle is also increased in the ASP^cKO^ tumors compared to ASP^WT^. However, no change in expression with neonatally expressed *Myh8* suggests ASP^cKO^ tumors resemble an earlier skeletal muscle progenitor (Fig. 2c). To better define the tumor cell differentiation state in the ASP^cKO^ and ASP^WT^ tumors, we bred *Rosa26*^*tdTomato*^ reporter mice into the ASP^WT^ and ASP^cKO^ models to generate ASTP mice. In the ASTP mice, tumor cells that have expressed aP2-Cre are indelibly labeled with tdTomato allowing tumor cell visualization. Immunofluorescent staining illustrated that nearly all tdTomato^+^ tumor cells in ASTP^WT^ mice expressed adult MyHC compared to only ~60% of the tdTomato^+^ tumor cells in ASTP^cKO^ mice (Fig. 2d, e). Furthermore, triple the number of tdTomato^+^ tumor cells in ASP^cKO^ mice were positive for MyHC-embryonic compared to ASP^WT^ mice while no change was observed for MyHC-perinatal (Fig. 2f, g and Supplementary Fig. 3a, b). These results further indicate that ASP^cKO^ tumors are less differentiated than ASP^WT^ tumors and more reflective of human ERMS.

### PTEN loss activates AKT but not mTORC1 in ASP^cKO^ tumors

**Fig. 3:**
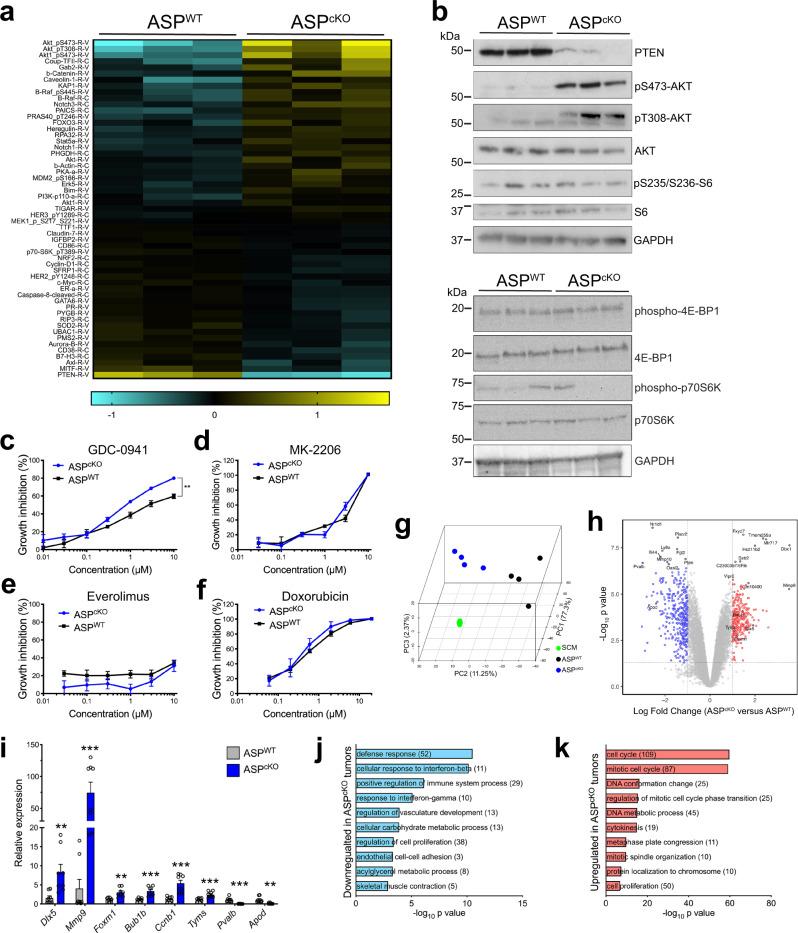
PTEN loss activates AKT but not mTORC1 in ASP^cKO^ tumors. **a** Heatmap of the 55 significantly (*P* < 0.05) different total and phosphoproteins by RPPA, normalized, log2 transformed and median-centered between ASP^WT^ and ASP^cKO^ tumors, *n* = 3. **b** Immunoblots of PTEN, phosphorylated AKT^Ser473^ or AKT^Thr308^, AKT, phosphorylated S6^Ser235/236^, S6, phosphorylated-4E-BP1, 4E-BP1, phosphorylated p70S6K, p70S6K, and GAPDH, *n* = 3. **c**–**f** CellTiterGlo growth assays of ASP^WT^ or ASP^cKO^ rhabdospheres treated with GDC-0941 (**c**) (*P* = 0.0020), MK-2206 (**d**), everolimus (**e**), or doxorubicin (**f**), *n* = 3, in triplicate. **g** Principal component analysis of transcriptional profiles from ASP^WT^ (black) and ASP^cKO^ (blue) tumors and Cre-negative SCM (green), *n* = 4. **h** Volcano plot showing differentially expressed genes between ASP^WT^ and ASP^cKO^ tumors from (**g**). Genes with increased (red) and decreased (blue) expression in ASP^cKO^ tumors (≥twofold, *P* < 0.05) are highlighted. **i** Validation of gene expression in (**h**) by real-time PCR (*n* = 3 SCM, *n* = 4 tumors, in duplicate), expression normalized to 18S rRNA and expressed relative to ASP^WT^. (*Dlx5* *P* = 0.0062, *Mmp9* *P* = 0.0009, *Foxm1* *P* = 0.0011, *Bub1b* *P* = 0.0002, *Ccnb1* *P* = 0.0002, *Tyms* *P* = 0.0009, *Pvalb* *P* = 0.0001, *Apod* *P* = 0.0018). **j**, **k** Gene ontology (GO) analyses of genes with increased (**j**) and decreased (**k**) expression in ASP^cKO^ tumors versus ASP^WT^ tumors. Top ten unique GO terms are shown. All *P* values in pairwise comparisons were determined by Student’s *t* test (unpaired, two-tailed); **P < 0.01, ***P < 0.001, ****P < 0.0001. Data represented as mean + SEM. See also Supplementary Figs. 4 and 5 and Supplementary Data 1. Source data are provided as a Source Data file.

Next, we sought to determine how PTEN loss affects cell signaling in the ASP^cKO^ tumors. PTEN loss and unchecked PI3K-pathway activation lead to aberrant activation/phosphorylation of kinases important in maintaining many hallmarks of cancer^11^. To determine the signaling pathways regulated by PTEN loss in ASP^cKO^ tumors, we performed an unbiased analysis of signaling proteins using reverse-phase protein arrays with 350 different antibodies directed against major cancer-related proteins. In ASP^cKO^ tumors, 30 probes were relatively overexpressed and 25 probes were relatively underexpressed (Fig. 3a). As expected, PTEN was the protein most decreased in ASP^cKO^ compared to ASP^WT^ tumors with concomitant increased AKT phosphorylation at both Thr308 and Ser473 (Fig. 3a, b). Among other proteins within the AKT/mTOR pathway, only phosphorylated PRAS40^Thr246^ was significantly relatively overexpressed in the ASP^cKO^ tumors. Surprisingly, the mTOR-downstream target phosphorylated p70S6K^Thr389^ was significantly decreased in ASP^cKO^ tumors, suggesting that AKT phosphorylation was largely uncoupled from mTORC1, further evidenced by lack of increased phosphorylation of S6 or 4E-BP1 (Fig. 3a, b). The other proteins increased in ASP^cKO^ tumors included both NOTCH1 and NOTCH3, constituents of MAPK cascades, and phosphorylated ERBB3 with its cognate ligand HEREGULIN. We confirmed increased ERK1/2 phosphorylation in ASP^cKO^ bulk tumor lysates (Supplementary Fig. 4a). However, we saw no increased sensitivity to trametinib in ASP^cKO^ primary rhabdospheres; this was probably due to a lack of maintained ERK1/2 phosphorylation observed in cultured ASP^cKO^ primary rhabdospheres (Supplementary Fig. 4b, c). Furthermore, immunosuppressive molecules B7-H3, CD86, and CD38 as well as necroptosome components cleaved caspase-8 and RIP3 (also known as RIPK3) were relatively underexpressed in ASP^cKO^ tumors suggesting less immune surveillance in the tumor microenvironment with *Pten* loss.

Given the increased AKT phosphorylation and absence of concomitant increased mTOR signaling in the ASP^cKO^ tumors, we examined whether these signaling results correlated with increased sensitivity to PI3K, mTOR, or AKT inhibitors. We dissected and dissociated ASP^WT^ and ASP^cKO^ tumors, seeded dissociated tumor cells to grow as rhabdospheres, and assayed cell growth when incubated with either GDC-0941 (pan-PI3K inhibitor), MK-2206 (AKT inhibitor), everolimus (mTOR inhibitor), or doxorubicin^20,24^. ASP^cKO^ rhabdospheres displayed increased sensitivity to GDC-0941 but no difference in sensitivity to MK-2206, everolimus, or doxorubicin (Fig. 3c–f). Although ASP^cKO^ tumors show increased phosphorylation of AKT, these results suggest the phenotypic differences observed in ASP^cKO^ tumors are most likely mediated through PI3K or alternative PTEN functions.

### *Dbx1* is the most overexpressed gene in ASP^cKO^ tumors and has a functional role in human FN-RMS

Given PTEN nuclear localization in ASP^WT^ tumors, we sought to determine if known nuclear functions were responsible for the ASP^cKO^ phenotype. Neither DNA damage nor chromosome instability was increased in the ASP^cKO^ tumors (Supplementary Fig. 5a–c). We next explored whether PTEN regulated gene expression and analyzed the gene expression changes in bulk ASP^WT^ and ASP^cKO^ tumors as well as SCM muscle. Principal component analysis revealed that gene expression in ASP^WT^ and ASP^cKO^ tumors clustered distinctly; however, they were more similar to each other than to mature SCM skeletal muscle (Fig. 3g and Supplementary Data 1). Comparing gene expression in ASP^cKO^ to ASP^WT^ tumors, 248 genes had increased and 286 genes had decreased expression (≥twofold, *P* value <0.05) (Fig. 3h). Several differentially expressed genes were validated by real-time PCR (Fig. 3i). Gene ontology analysis of the 286 genes with decreased expression in ASP^cKO^ tumors compared to ASP^WT^ tumors uncovered gene ontology (GO) terms associated with skeletal myogenic differentiation consistent with increased expression of embryonic MyHC and decreased expression of adult MyHC in ASP^cKO^ tumors, and dysregulated immune responses (Figs. 2d–g and 3j); conversely, 248 genes with increased expression (Supplementary Data 1) revealed GO terms associated with increased proliferation and DNA replication consistent with increased Ki67 staining in ASP^cKO^ tumors (Figs. 1e, f and 3k). Furthermore, we utilized CIBERSORT to analyze gene expression profiles from ASP^WT^ and ASP^cKO^ tumors to predict immune cell composition; ASP^cKO^ tumors were predicted to have less naive B cells, M1 macrophages, resting mast cells, and eosinophils while M0 macrophages are enriched in the ASP^cKO^ tumors (Supplementary Fig. 4d).

**Fig. 4:**
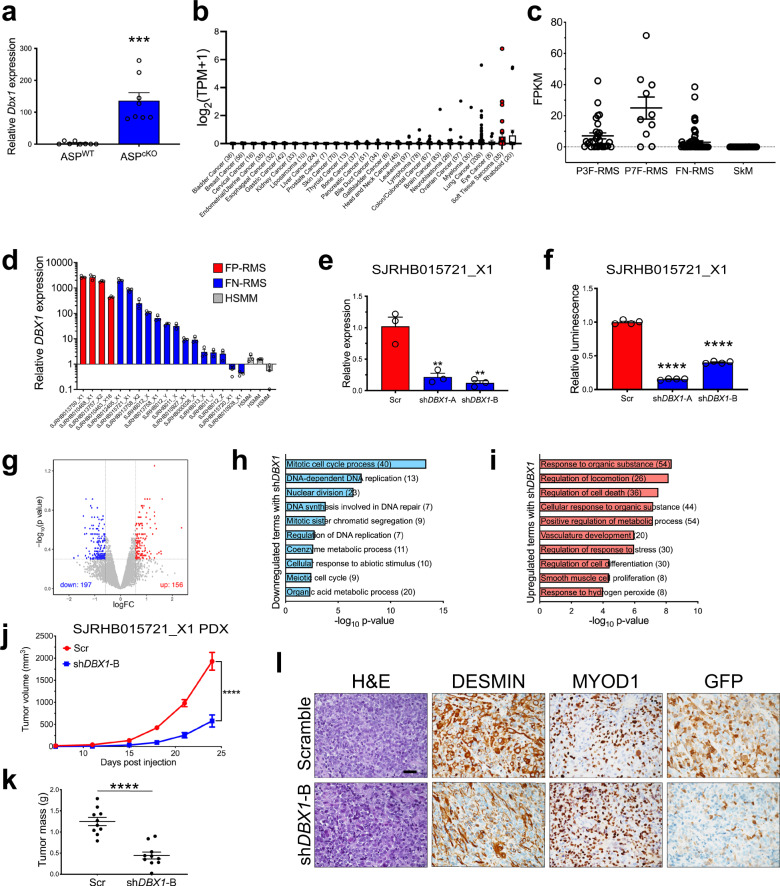
*Dbx1* is the most overexpressed gene in ASP^cKO^ tumors and has a functional role in human FN-RMS. **a** Gene expression by real-time PCR of *Dbx1* (*P* = 0.0001) in ASP^WT^ tumors (*n* = 4) ASP^cKO^ tumors (*n* = 4), in duplicate. **b** *DBX1* mRNA expression (log_2_(TPM + 1)) in cell lines from the Broad Institute Cancer Dependency Map (DepMap), 20Q3 release. Soft-tissue sarcoma cell lines are in red. The number of cell lines is indicated along the *x* axis label for each respective tumor type. **c** *DBX1* expression by RNA sequencing (FPKM) in human FN-RMS (*n* = 64), FP-RMS (PAX3-FOXO1 (P3F, *n* = 29) and PAX7-FOXO1 (P7F, *n* = 10)) tumors, and human skeletal muscle samples and human myoblast tissue cultures (*n* = 26) (SkM) from the NCI^30^. **d** Gene expression by real-time PCR of *DBX1* in RMS PDXs and human skeletal muscle myoblasts (HSMMs), in triplicate. **e** Gene expression by real-time PCR of *DBX1* in SJRHB015721_X1 cell line following lentiviral transduction (72 h) with *DBX1*-targeting or control shRNA (Scr), in triplicate. (Scr-sh*DBX1-*A *P* = 0.0068, Scr-sh*DBX1-*B *P* = 0.0039). **f** Cell viability by Cell Titer Glo assay of SJRHB015721_X1-derived adherent cell cultures transduced with *DBX1-*targeting or control shRNAs after 5 days; in quadruplicate, compared to scramble. (Scr-sh*DBX1-*A *P* < 0.0001, Scr-sh*DBX1-*B *P* < 0.0001). **g** Volcano plot depicting differentially expressed genes between two *DBX1*-targeted and control shRNAs (absolute log fold change >0.58, −log_10_(adjusted *P* < 0.5) in SJRHB015721_X1 cells. **h**, **i** GO analyses of genes with decreased (**h**) and increased (**i**) expression following shRNA-mediated *DBX1* depletion in the SJRHB015721_X1 cell line. Top ten unique GO terms are shown, DAVID analysis. **j**, **k** Tumor volume of SJRHB015721_X1 orthotopic patient-derived xenograft (PDX) tumor cells transduced with *DBX1*-targeting or control shRNAs (**j**, *P* < 0.0001) and tumor mass (**k**, *P* < 0.0001), *n* = 10. **l** Representative H&E staining and IHC for DESMIN, MYOD1, and GFP from tumors in (**j**). Scale bar = 25 μm. All *P* values in pairwise comparisons were determined by Student’s *t* test (unpaired, two-tailed); **P < 0.01, ****P < 0.0001. Data represented as mean + SEM. Real-time PCR normalized to 18S rRNA and expressed relative to ASP^WT^ (**a**), the average of the HSMM cell lines (**d**), or scramble (**e**). See Supplementary Fig. 6 and Supplementary Data 1 and  2. Source data are provided as a Source Data file.

The subset of genes relatively overexpressed in ASP^cKO^ tumors included neuronal transcription factors including *Dlx5* and *Pax7* as well as *Dbx1* (*Developing brain homeobox 1*), the most overexpressed gene in the ASP^cKO^ tumors (Fig. 4a). DBX1 is a homeobox transcription factor thought to function as a transcriptional repressor critical in neural patterning of innate processes^25–27^. However, the role of DBX1 in cancer is unknown.

Given the lack of knowledge about DBX1’s function in cancer, we first interrogated the Cancer Dependency Map^28^ and found that *DBX1* highly expressed in RMS cell lines with only rhabdoid tumors exhibiting higher expression (Fig. 4b). Next, we probed two public human RMS expression datasets both illustrating increased *DBX1* expression in FN-RMS (Fig. 4c and Supplementary Fig. 6a)^29,30^. Furthermore, increased *DBX1* expression was also seen in all FP-RMS and most FN-RMS patient-derived xenografts (PDX) from the Childhood Solid Tumor Network at St. Jude compared to primary human myoblasts (Fig. 4d)^31^. Therefore, DBX1 expression is increased in both mouse and human FN-RMS; however, the functional significance of DBX1 in RMS biology remains unknown.

To study the role of DBX1 in human FN-RMS, we generated a cell line from FN-RMS PDX SJRHB015721_X1 with high *DBX1* expression (Fig. 4d and Supplementary Fig. 6b, c). We transduced the cells with lentivirus expressing either two independent shRNAs targeting *DBX1* or a scramble shRNA control. *DBX1-*targeting shRNAs decreased *DBX1* expression and cell viability compared to the scramble control, suggesting a role for *DBX1* in FN-RMS (Fig. 4e, f). To further understand the functional role of DBX1 in RMS, we performed gene expression analysis on the SJRHB015721_X1 cell line transduced with the two shRNAs and scramble control. 197 genes had decreased expression and 156 genes had increased expression in both shRNAs compared to scramble control (absolute log fold change >0.58, −log_10_ adjusted *P* < 0.5) (Fig. 4g and Supplementary Fig. 6d and Supplementary Data 2). Gene ontology analysis of the 197 genes with decreased expression with *DBX1* knockdown highlights processes related to cell proliferation (Fig. 4h and Supplementary Data 2). The GO terms of the 156 genes with increased expression upon *DBX1* knockdown were enriched for apoptotic processes as well as positive regulators of development and morphogenesis (Fig. 4i and Supplementary Data 2). These data suggest the DBX1 corepressor activity assists in maintaining the proliferative state and blocked differentiation in FN-RMS.

To determine the in vivo significance of DBX1, we dissociated two human FN-RMS PDX samples, SJRHB015721_X1 and SJRHB012405_X1, and transduced cells with lentiviral vectors expressing EGFP as a marker of transduction and shRNA to knockdown *DBX1* expression. After transduction, cells were injected into the gastrocnemius of SCID/Beige mice and used to establish primary rhabdosphere cultures. *DBX1* expression decreased with shRNA expression in rhabdospheres indicating efficient *DBX1* knockdown after viral transduction (Supplementary Fig. 6e, f). *DBX1* knockdown in both SJRHB015721_X1 and SJRHB012405_X1 PDXs reduced tumor volume and tumor mass (Fig. 4j, k and Supplementary Fig. 6g, h, j, k). Gross and immunohistochemical analysis indicated that the DBX1 knockdown tumors were indistinguishable from the scramble controls except that the sh*DBX1* tumors that formed lost EGFP expression (Fig. 4l and Supplementary Fig. 6i, l). This suggests that tumors developing from shDBX1-transduced cells had escaped lentiviral transduction and thus escaped *DBX1* knockdown highlighting the importance of DBX1 in FN-RMS.

### PAX7 controls *Dbx1* expression

**Fig. 5:**
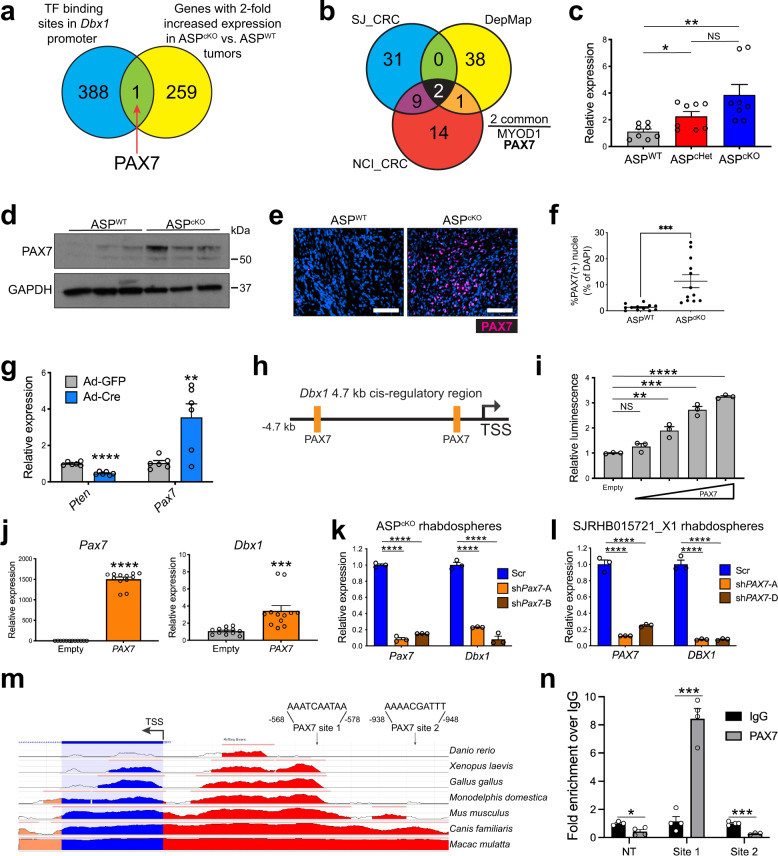
PAX7 controls *Dbx1* expression. **a** Venn diagram depicting *Pax7* as the sole overlapping gene between transcripts enriched in ASP^cKO^ tumors and transcription factors (TFs) with putative binding sites (from JASPAR 2016)^72^ within the *Dbx1* promoter (1 kb upstream of TSS). **b** Venn diagram depicting the overlap between core transcriptional circuitry genes and FN-RMS DepMap (Cancer Dependency Map) gene dependencies^28,31,35^. **c** Gene expression by real-time PCR of *Pax7* in ASP^WT^ and ASP^cKO^ tumors. Expression normalized to *Actb* and expressed relative to ASP^WT^, *n* = 4, in duplicate. (ASP^WT^-ASP^cHet^ *P* = 0.0123, ASP^WT^-ASP^cKO^ *P* = 0.0046). **d** Immunoblot of PAX7 and GAPDH in ASP^WT^ and ASP^cKO^ tumors lysates, *n* = 3. **e**, **f** Representative IF depicting increased PAX7 (red) staining in ASP^cKO^ tumors versus ASP^WT^ tumors (DAPI nuclear stain, blue) (**e**) quantitated in (**f**), *n* = 3, 12 fields of view per genotype, *P* = 0.0006. Scale bar = 25 μm. **g** Gene expression by real-time PCR of *Pax7* and *Pten* following 72 h post-adenoviral-Cre infection of *Pten*^flox/flox^ mouse embryonic fibroblasts (MEFs). Expression normalized to 18S rRNA and expressed relative to Ad-GFP-infected cells, *n* = 3 independent MEF cultures, in duplicate. (*Pten* *P* < 0.0001, *Pax7* *P* = 0.0080). **h** Cartoon illustrating putative PAX7-binding sites in the 4.7 kb *Dbx1* *cis*-regulatory region upstream of transcription start site (TSS). **i** Representative *Dbx1*-promoter-luciferase assay depicting a PAX7 dose-dependent increase in luciferase activity. *n* = 3 independent assays, in technical triplicate. (empty 250 ng *P* = 0.0043, empty 500 ng *P* = 0.0002, empty 750 ng *P* < 0.0001). **j** Gene expression by real-time PCR of *Dbx1* (*P* = 0.0009) and *Pax7* (*P* < 0.0001) following stable transduction of wild-type MEFs with *Pax7*. Expression normalized to 18S rRNA and relative to empty control, *n* = 6 independent MEF cultures, in duplicate. **k** Gene expression by real-time PCR of *Dbx1* and *Pax7* in primary ASP^cKO^ rhabdospheres transduced with *Pax7*-targeting or scramble shRNAs, 5 days post transduction, in triplicate. Normalized to *Actb* expression and expressed relative to scramble. All *P* values <0.0001. **l** Gene expression by real-time PCR of *DBX1* and *PAX7* in SJRHB015721_X1 patient-derived rhabdospheres transduced with *PAX7* targeting or control harvested 8 days post transduction, in triplicate. Normalized to 18S expression and expressed relative to scramble. All *P* values <0.0001. **m** Evolutionary conservation of the human *DBX1*-promoter region (1552 bp depicted from within the first intronic region through first 1000 bp upstream of the DBX1 transcription start site) as obtained from the Evolutionary Conserved Regions database (ecrbrowser.dcode.org)^71^. Pink bars on top of each species track show conservation to the human sequence. Salmon coloring indicates conserved intronic regions, blue indicates conserved protein-coding sequences, and red indicates conserved intergenic regions. Also depicted are the two putative PAX7-binding sites (with binding sequence) from the JASPAR^73^ database within the *DBX1*-promoter sequence. All species compared to the human *DBX1* DNA sequence. **n** ChIP-PCR of PAX7 binding to the proximal site 1 and not distal site 2 within the *DBX1* promoter in SJRHB015721_X1 cells. NT = non-PAX7-binding amplicon within the *DBX1* promoter; two biologically independent samples performed in duplicate. Normalized to IgG binding at respective sites. (NT *P* = 0.0074, site 1 *P* = 0.0001, site 2 *P* = 0.0003). All *P* values in pairwise comparisons were determined by Student’s *t* test (unpaired, two-tailed); *P < 0.05, **P < 0.01, ***P < 0.001, ****P < 0.0001. Data represented as mean + SEM. Source data are provided as a Source Data file.

Given *Dbx1* is the most overexpressed gene in ASP^cKO^ tumors and *DBX1* knockdown decreased tumor growth in human FN-RMS cells, we sought to determine how *Pten* loss regulates *Dbx1* expression. We first examined a 1 kb promoter region immediately 5’ of *Dbx1*’s initiating ATG to identify known transcription factor binding motifs and compared this to genes with >twofold expression in ASP^cKO^ tumors and found only one overlapping gene – *Pax7* (Fig. 5a). PAX7 is a transcription factor critical in both adult muscle stem cell (satellite cells) maintenance and preserving a dedifferentiated state in FN-RMS^32,33^. Interestingly, PAX7 along with MYOD1 are the only genes that are both members of FN-RMS core-regulatory circuits and are FN-RMS dependencies thus coupling tumor cell identity and survival (Fig. 5b)^34,35^. We confirmed increased *Pax7* expression in ASP^cKO^ tumors compared to ASP^WT^ tumors by real-time PCR, immunoblotting, and immunofluorescence (Fig. 5c–f). To confirm the link between PTEN and *Pax7* transcription, we infected *Pten*^*flox*^ mouse embryonic fibroblasts (MEFs) with adenovirus encoding either Cre recombinase or GFP. Adeno-Cre-infected *Pten*^*flox*^ MEFs exhibited decreased *Pten* expression and increased *Pax7* (Fig. 5g). Next, to determine if PAX7 transcriptionally regulates *Dbx1*, we cloned 900 bp of the *Dbx1* promoter containing a putative PAX7-binding site upstream of luciferase. PAX7 expression led to a dose-dependent increase in *Dbx1*-promoter-driven luciferase activity (Fig. 5h, i). Next, we transduced wild-type MEFs with lentivirus overexpressing PAX7 and showed concomitant increased *Dbx1* expression (Fig. 5j). Conversely, shRNA knockdown of *Pax7* in ASP^cKO^ rhabdospheres resulted in decreased *Dbx1* expression (Fig. 5k). Furthermore, decreased *PAX7* expression in human rhabdospheres led to decreased *DBX1* expression (Fig. 5l).

The murine and human DBX1 proximal promoters are highly conserved (Fig. 5m). Of the two putative PAX7-binding sites within the human *DBX1* promoter, only the proximal (site 1) is conserved in the mouse. We designed primers that flank each of these human sites and performed chromatin immunoprecipitation (ChIP)-qPCR to analyze whether PAX7 is bound to the *DBX1* promoter. PAX7 binding was enriched eightfold over control IgG at the proximal *DBX1* promoter PAX7-binding motif (which also corresponds to the mouse *Dbx1* promoter) (Fig. 5n). These data indicate PTEN loss increases PAX7 expression and DBX1 is a downstream target of PAX7 in FN-RMS.

### Human FN-RMS is dependent on PAX7

**Fig. 6:**
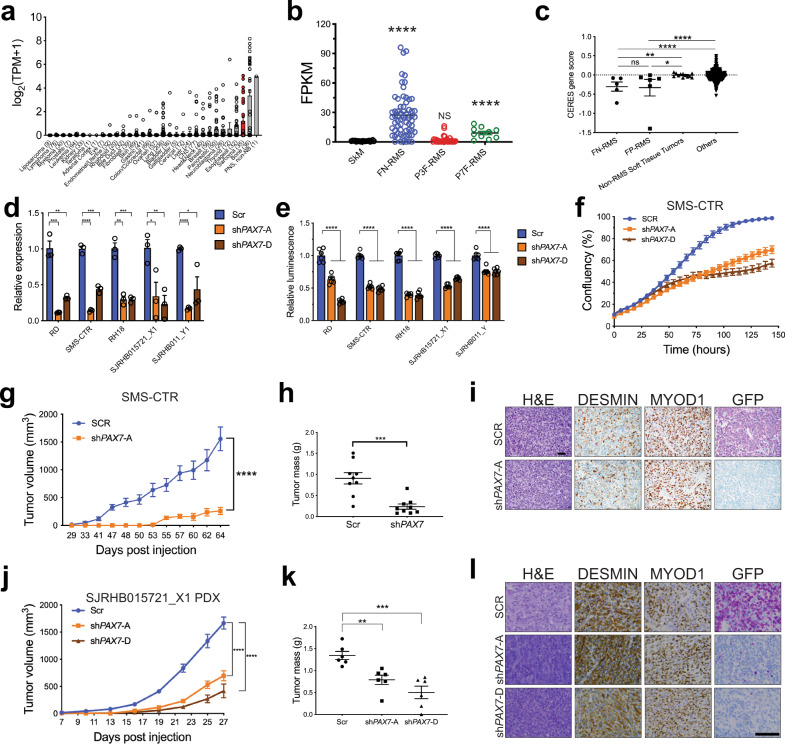
Human FN-RMS is dependent on PAX7. **a***PAX7* expression by RNA sequencing (log_2_(TPM + 1)) from the DepMap (20Q3 release). Red bar indicates sarcoma cell lines. The number of cell lines is indicated along the *x* axis label for each respective tumor type. **b** RNA sequencing (FPKM) of *PAX7* in human FN-RMS (*n* = 64), FP-RMS (P3F-RMS *n* = 29, P7F- RMS *n* = 10) tumors, and human skeletal muscle (*n* = 20) (SkM) from the NCI dataset^30^. (SkM-FN-RMS *P* < 0.0001, SkM-P7F-RMS *P* < 0.0001). **c** CERES gene dependency score for *PAX7* in FN-RMS cells (*n* = 5) compared to all other cell lines in the DepMap, 20Q3 release. (FN-RMS-non-RMS soft-tissue tumors *P* = 0.0015, FP-RMS-non-RMS soft-tissue tumors *P* = 0.0450, FN-RMS-others and FP-RMS-others *P* < 0.0001). **d** Gene expression by real-time PCR of *PAX7* in SMS-CTR, RD, Rh18, SJRHB015721_X1, and SJRHB011_Y1-cultured cells following transduction with *PAX7* targeting or scramble shRNA, in triplicate, 5 days post transduction. Normalized to *ACTB* expression and expressed relative to scramble. (RD Scr-sh*PAX7-*A *P* = 0.008, RD Scr-sh*PAX7-D* *P* = 0.0023) (SMS-CTR Scr-sh*PAX7-*A *P* < 0.0001 Scr-sh*PAX7-*D *P* = 0.0003) (Rh18 Scr-sh*PAX7*-A *P* = 0.0013, Scr-sh*PAX7*-D *P* = 0.0009) (SJRHB015721_X1 Scr-sh*PAX7-*A *P* = 0.0406, Scr-sh*PAX7*-D *P* = 0.0092) (SJRHB011_Y1 Scr-sh*PAX7-*A *P* < 0.0001, Scr-sh*PAX7*-D *P* = 0.0318). **e** Cell viability by Cell Titer Glo assay of RD, SMS-CTR, Rh18, SJRHB015721_X1, and SJRHB011_Y cell lines after *PAX7* knockdown, in quadruplicate. All *P* values are <0.0001. **f** SMS-CTR proliferation assay following *PAX7* knockdown, *n* = 30 images (six wells, five images taken per well). **g**–**i** Tumor volume over time (**g**, *P* < 0.0001) and final tumor mass (**h**, *P* = 0.0003) of orthotopically injected SMS-CTR cells in SCID/Beige mice (*n* = 10 per group) transduced with *PAX7* targeting or scramble shRNAs. **i** representative histology of tumors from (**g**), *n* = 10. **j**–**l** Tumor volume over time (**i**, all *P* values <0.0001) and final tumor mass (**j**, Scr-sh*PAX7-*A *P* = 0.0028, Scr-sh*PAX7*-D *P* = 0.0006) of orthotopically injected SJRHB015721_X1 cells in SCID/Beige mice transduced with *PAX7* targeting or scramble shRNAs, *n* = 6. **l** representative histology of tumors from (**j**), *n* = 6. All *P* values in pairwise comparisons were determined by Student’s *t* test (unpaired, two-tailed); *P < 0.05, **P < 0.01, ***P < 0.001, ****P < 0.0001. Data represented as mean + SEM. See also Supplementary Fig. 7. Source data are provided as a Source Data file.

Given that PAX7 is putatively both a transcription factor in the RMS core-regulatory circuit and a dependency in RMS, we sought to further explore the role of PAX7 in RMS. The Broad Institute’s Cancer Dependency Map (DepMap) illustrates that *PAX7* is highly expressed in soft-tissue sarcoma cell lines (Fig. 6a)^28^. Specifically, *PAX7* expression is increased in human FN-RMS tumors compared to skeletal muscle in a public human RMS expression dataset (Fig. 6b)^30^, as well as in human FN-RMS cell lines and PDXs compared to myoblasts (Supplementary Fig. 7a, b). CRISPR/Cas9-dependency screening in DepMap revealed a PAX7 dependency in FN-RMS cell lines (Fig. 6c). Thus, we wanted to confirm if human FN-RMS cell lines and PDXs are dependent on PAX7 expression. Using lentiviruses expressing two independent shRNA targeting *PAX7* or a scramble control, we performed *PAX7* knockdown in human FN-RMS cell lines (SMS-CTR, RD, Rh18) and cell lines derived from two PDXs – SJRHB015721_X1 and SJRHB011_Y (Fig. 6d and Supplementary Fig. 7c, d). *PAX7* knockdown in FN-RMS cells decreased both cell viability and cell growth in vitro suggesting a role in FN-RMS (Fig. 6e, f and Supplementary Fig. 7e–h).

Since *PAX7* knockdown reduced FN-RMS cell proliferation in vitro, we aimed to determine whether sh*PAX7* knockdown affected tumorigenicity in vivo. SMS-CTR cells transduced with sh*PAX7* or scramble control were injected into the gastrocnemius of SCID/Beige mice. *PAX7* knockdown significantly reduced tumor volume and mass in SMS-CTR cell xenografted mice (Fig. 6g, h, i). To establish this was not a consequence or artifact of using an established cell line, we extended our analysis to FN-RMS PDXs. SJRHB015721_X1 and SJRHB012405_X1 PDX cells were dispersed into single cells and transduced with lentivirus expressing sh*PAX7* or scramble control. After a brief incubation at 37 °C, the cells were either placed in spheroid culture or injected into the gastrocnemius muscle of SCID/Beige mice. *PAX7* knockdown was confirmed in spheroid culture cells (Supplementary Fig. 7i). In both SJRHB015721_X1 and SJRHB012405_X1 PDXs, *PAX7* knockdown with sh*PAX7* resulted in decreased tumor volume (Fig. 6j and Supplementary Fig. 7j); however, tumor mass only decreased in SJRHB015721_X1 (Fig. 6k and Supplementary Fig. 7k). Tumors from SJRHB012405_X1 retained *PAX7* expression at the endpoint suggesting a negative selection for cells with *PAX7* knockdown (Fig. 6l and Supplementary Fig. 7l). Together, our in vitro and in vivo data confirm *PAX7* is a dependency in FN-RMS cells.

### PAX7 dictates rhabdomyosarcoma tumor cell identity in ASP^cKO^ tumors

**Fig. 7:**
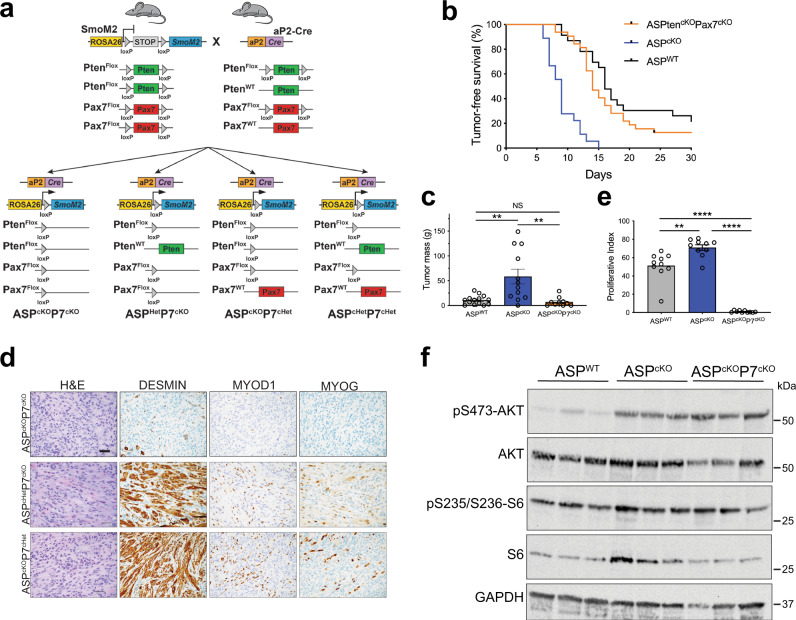
PAX7 loss rescues deleterious effects of *Pten* loss in ASP^cKO^ tumors. **a** Breeding strategy to generate ASP^cKO^P7^cKO^ tumor mice. **b** Kaplan–Meier tumor-free survival curve of ASP^cKO^P7^cKO^ (*n* = 32) in comparison to ASP^WT^ and ASP^cKO^ mice (from Fig. 1b). Mantel–Cox log rank, *P* < 0.0001. **c** Tumor mass of ASP^cKO^P7^cKO^ (*n* = 10), ASP^WT^ (*n* = 11) and ASP^cKO^ (*n* = 12) tumors at P12. ASP^WT^ and ASP^cKO^ data are the same from Fig. 1c. (ASP^WT^-ASP^cKO^ *P* = 0.0022, ASP^cKO^-ASP^cKO^P7^cKO^ *P* = 0.0056). **d** Representative histology of ASP^cKO^P7^cKO^ (*n* = 14), ASP^cHet^P7^cKO^ (*n* = 4), and ASP^cKO^P7^cHet^ (*n* = 4) tumors by H&E staining and IHC for DESMIN, MYOD1, and MYOG. Scale bar, 25 μm. **e** Quantitation of percent Ki67-positive nuclei in ASP^WT^ (*n* = 9), ASP^cKO^ tumors (*n* = 7), and ASP^cKO^P7^cKO^ (*n* = 5) tumors (ten random fields of view per genotype). ASP^WT^ and ASP^cKO^ from Fig. 1f. (ASP^WT^-ASP^cKO^ *P* = 0.0029, ASP^WT^-ASP^cKO^P7^cKO^ *P* < 0.0001, ASP^cKO^-ASP^cKO^P7^cKO^ *P* < 0.0001). **f** Immunoblots of phosphorylated AKT^Ser473^, AKT, phosphorylated S6^Ser235/236^, S6, and GAPDH, *n* = 3. All *P* values in pairwise comparisons were determined by Student’s *t* test (unpaired, two-tailed); NS   not significant, **P < 0.01, ****P < 0.0001. Data represented as mean + SEM. Source data are provided as a Source Data file.

To elucidate the mechanistic role of PAX7 in FN-RMS, we returned to our ASP^cKO^ mouse model. We bred *Pax7*^flox^ alleles into the ASP^cKO^ model to generate compound conditional knockout mice, *aP2-Cre;Smo*^*M2/+*^*;Pten*^*flox/flox*^*Pax7*^*flox/flox*^ (ASP^cKO^P7^cKO^) and associated littermate control mice (Fig. 7a). Deleting *Pax7* in ASP^cKO^ mice rescued the decreased tumor-free survival and increased tumor mass associated with *Pten* loss in the AS mice (Fig. 7b, c). ASP^cKO^P7^cKO^ tumors were noncircumscribed and highly infiltrative with the tumor cells perpendicular to and through the skeletal muscle fibers (Fig. 7d). Histologically, the ASP^cKO^P7^cKO^ tumor cells stained sporadically for DESMIN but were no longer positive for RMS diagnostic markers MYOD1 and MYOGENIN; this was specific for ASP^cKO^P7^cKO^ tumors as ASP^cHet^P7^cKO^ and ASP^cKO^P7^cHet^ tumors were still myogenic, small round blue tumors consistent with RMS (Fig. 7d). The ASP^cKO^P7^cKO^ tumors were also significantly less proliferative than either ASP^WT^ and ASP^cKO^ tumors (Fig. 7e). However, these phenotypes cannot be attributed to differences in PI3K-pathway activation observed in ASP^cKO^ and ASP^WT^ tumors as ASP^cKO^P7^cKO^ tumors had similar AKT and S6 phosphorylation as ASP^cKO^ tumors (Fig. 7f). This highlights a unique link between PTEN and PAX7 independent of PI3K-pathway signaling that illustrates PAX7 is a synthetically essential gene in PTEN-deficient FN-RMS.

**Fig. 8:**
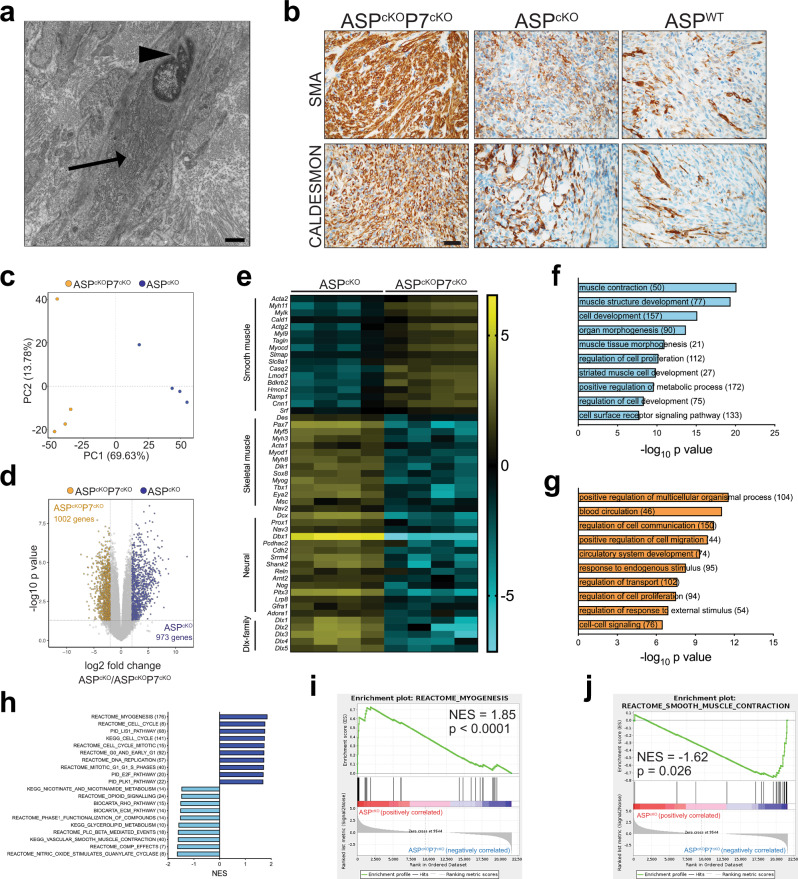
PAX7 dictates rhabdomyosarcoma identity in ASP^cKO^ tumors. **a** Representative transmission electron micrograph of an ASP^cKO^P7^cKO^ tumor (*n* = 3) showing hallmarks of LMS; arrowhead = grooved nuclei, and arrow = prominent rough endoplasmic reticulum. Scale bar = 1 μm. **b** Representative histology of smooth-muscle differentiated tumor markers SMA and CALDESMON in ASP^cKO^P7^cKO^ (*n* = 14), ASP^cKO^ (*n* = 4), and ASP^WT^ (*n* = 4) tumors. Scale bar, 25 μm. **c**, **d** Principal component analysis (**c**) and a volcano plot (**d**) of top 3000 differentially expressed genes (for c) and the significantly different (*P* < 0.05) and fourfold differentially expressed genes between the flow cytometry-sorted ASP^cKO^ and ASP^cKO^P7^cKO^ tumor cells (**d**). Data represented as the log2 fold change of ASP^cKO^/ASP^cKO^P7^cKO^. Gold indicates the 1002 genes enriched in the ASP^cKO^P7^cKO^ tumor cells. Blue indicates the 973 genes enriched in the ASP^cKO^ tumor cells. Complete RNAseq data shown in Supplementary Data 3. *P* values calculated by two-tailed unpaired *t* test in limma package. **e** Heatmap of selected significantly (*P* < 0.05) differentially expressed genes (>fourfold, mean centered per gene) between tdTomato^+^/CD31^−^ tumor cell populations from ASTP^cKO^ and ASTP^cKO^P7^cKO^ tumors, *n* = 4 tumors per genotype. **f**, **g** GO analysis of differentially expressed genes (up in ASP^cKO^, (**f**)) (up in ASP^cKO^P7^cKO^, (**g**)) between flow cytometry-sorted tumor populations from both genotypes; top ten unique GO terms depicted following DAVID functional annotation clustering. **h** Normalized enrichment scores of gene sets enriched ASP^cKO^ tumors compared to ASP^cKO^P7^cKO^ tumors according to Gene Set Enrichment Analysis (GSEA). Dark blue = enriched in ASP^cKO^ and light blue = enriched in ASP^cKO^P7^cKO^. **i**, **j** Enrichment plots of skeletal myogenic (**i**, Reactome, myogenesis) and smooth muscle (**j**, Reactome, smooth-muscle contraction) gene signatures from (**h**). See also Supplementary Fig. 8 and Supplementary Data 3. Source data are provided as a Source Data file.

To determine the tumor type produced following concomitant *Pten* and *Pax7* depletion, we first performed ultrastructural analysis and that revealed ASP^cKO^P7^cKO^ tumor features were no longer consistent with skeletal muscle but with leiomyosarcoma (LMS), including elongated cellular shape, grooved nuclei, and prominent rough endoplasmic reticulum (Fig. 8a). Intriguingly, ASP^cKO^P7^cKO^ tumors but not ASP^WT^ or ASP^cKO^ tumors expressed the LMS markers smooth-muscle actin (SMA) and CALDESMON (Fig. 8b). Given that ASP^cKO^P7^cKO^ tumors had a lower proliferative index (Fig. 7e) and did not engraft into SCID/Beige immunocompromised mice (0/6 tumors), the tumors that arise may lie along a spectrum of low-grade sarcomas with smooth-muscle immunophenotypic features. These results illustrate that the *Pten* loss phenotype is dependent on PAX7 and indicates that PAX7 controls FN-RMS cell fate.

To explore the mechanisms altering ASP^cKO^P7^cKO^ tumor identity, we compared the gene expression in ASP^cKO^ and ASP^cKO^P7^cKO^ tumors. We bred the *Rosa26*^tdTomato^ reporter mouse into our ASP^cKO^P7^cKO^ model to generate ASTP^cKO^P7^cKO^ compound mutant mice allowing us to use flow cytometry to purify tumor cells by sorting tdTomato-positive and CD31 (PECAM)-negative (to remove endothelial cells) cells (Supplementary Fig. 8a)^20^. RNAseq from sorted Tom^+^CD31^−^ tumor populations revealed robust differential gene expression with 973 genes enriched in ASTP^cKO^ and 1002 genes enriched in ASTP^cKO^P7^cKO^ tumor cells (≥twofold, *P* < 0.05) (Fig. 8c, d and Supplementary Data 3). Functional annotation clustering and gene set enrichment analyses further highlighted differences between ASTP^cKO^ and ASTP^cKO^P7^cKO^ tumors with myogenic and neurogenic transcriptional programs enriched in ASTP^cKO^ and relatively underexpressed in ASTP^cKO^P7^cKO^ (Fig. 8e, f, h, i and Supplementary Data 3). This dual regulation of skeletal myogenic and neurogenic genes is important to note as PAX7 is a transcription factor that regulates both developmental programs^36,37^. Curiously, all five distal-less homeobox genes were also relatively underexpressed in ASTP^cKO^P7^cKO^, indicating PAX7 as a master regulator of their expression (Fig. 8e).

In contrast to the increase in skeletal myogenic and neurogenic transcriptional programs enriched in the ASTP^cKO^ tumors, there were relatively few transcriptional programs increased in ASTP^cKO^P7^cKO^ tumors (Fig. 8e, g, h, j and Supplementary Data 3). The major pathways enriched in ASTP^cKO^P7^cKO^ tumors included calcium signaling, cyclic nucleotide pathways, and most intriguingly, vascular smooth muscle including the smooth muscle master-regulator transcription factor Myocardin (*Myocd*)^38^. Furthermore, ASTP^cKO^P7^cKO^ tumors were enriched for genes associated with LMS, including smooth-muscle structural proteins *Acta2* and *Myh11*. Most of the LMS genes (*Lmod1*, *Casq2*, *Actg2*, *Mylk*, and *Slmap*) are markers of subtype I of human LMS which is mostly found in extrauterine sites^39^. Unlike the expression differences in *Lmod1*, *Myh11*, and *Acta2* observed between ASP^cKO^P7^cKO^ and ASP^cKO^ tumors (Supplementary Fig. 8b), we did not find any comparable increases in *LMOD1*, *MYH11*, and *ACTA2* following shRNA-mediated knockdown of PAX7 expression in three human FN-RMS cell lines/PDXs—SMS-CTR, SJRHB015721_X1, and SJRHB012405_X1—highlighting that this PAX7-controlled fate decision occurs early in tumorigenesis (Supplementary Fig. 8c–e). Furthermore, tumors that form in xenografts of SMS-CTR cell line and the SJRHB015721_X1 and SJRHB12405_X1 PDXs following *PAX7* knockdown have not shifted fate to LMS by IHC for SMA and CALDESMON (Supplementary Fig. 8f). These results indicate that deletion of *Pax7* in the ASP^cKO^ murine FN-RMS model yields tumors whose histology, ultrastructure, and gene expression recapitulates low-grade sarcoma with smooth-muscle differentiation and not a skeletal myogenic tumor-like FN-RMS. Therefore, simultaneous *Pten* and *Pax7* loss in murine FN-RMS not only rescued the effects of *Pten* loss in these tumors but identified a synthetic essential relationship between *Pten* and *Pax7* controlling tumor cell fate.

## Discussion

The cell of origin and genetic perturbations cooperate in tumor cell transformation and fate determination highlighting cellular and developmental pliancy in tumorigenesis. Genetically engineered mouse models offer a method to dissect these relationships by utilizing *aP2-Cre* to activate oncogene and/or delete tumor suppressor expression. Genetic fate mapping reveals aP2-Cre expression in white and brown adipocytes, dorsal root ganglia, macrophages, and endothelial cells^20,22,40^. Compound mutant mice using aP2-Cre to drive recombination resulting in biallelic *Dicer1* deletion or the combination of oncogenic *Kras*^G12D^ activation and *Cdkn2a* deletion result in angiosarcoma from the transformation of endothelial cells^20,41^. However, activation of constitutively active, oncogenic SMO^M2^, and hedgehog pathway signaling by aP2-Cre results in cell reprogramming and transformation of endothelial progenitor cells driving FN-RMS tumorigenesis^20^. We now find *Pten* loss cooperates with SMO^M2^ in aP2-Cre expressing cells to drive a less differentiated tumor that better recapitulates the most common human embryonal RMS histology. This was specific to *Pten* loss as *Cdkn2a*, *Rb1*, or *Trp53* loss did not phenocopy the results. The effects of *Pten* deletion were dependent on PAX7 as *Pax7* deletion rescued the phenotypes of *Pten* loss and altered tumor cell fate resulting in a spectrum of low-grade sarcomas with immunophenotypic smooth-muscle differentiation, including LMS. Interconnections between RMS and LMS have been described with anecdotal clinical reports of LMS co-occurring with RMS or with rhabdomyosarcomatous-like lesions^42,43^. This suggests there may be some shared developmental trajectories and highlights the fluidity of cellular and developmental pliancy in tumorigenesis.

*Dbx1* was the most relatively overexpressed gene in ASP^cKO^ tumors and has not been reported to have a role in cancer. DBX1 is an evolutionarily conserved homeobox transcription factor expressed embryonically in central nervous tissue functioning as a transcriptional corepressor to establish specific neuron fate and neural patterning^25–27^. We found that both human FN-RMS and FP-RMS had subsets of tumor with high *DBX1* expression. Interestingly, DBX1 transcription was controlled by the skeletal muscle satellite cell marker PAX7 in both murine and human FN-RMS models. Since FP-RMS tumors are driven by PAX3-FOXO1 or PAX7-FOXO1 fusion oncoproteins containing either PAX3 or PAX7 DNA binding domains, we postulate that *DBX1* expression is driven transcriptionally by the fusion oncoproteins much how PAX7 itself targets DBX1 expression in FN-RMS. This may suggest some shared etiology between two major RMS subtypes.

Importantly, PAX7 not only functions in skeletal muscle development and maintenance of satellite cell quiescence but also other cell types, including pituitary gland melanotropes, neural tube, and actively migrating neural crest cells^36,37,44,45^. ASP^cKO^ tumors engage a transcriptional program composed of several neural-associated genes, many of which are under the control of PAX7 as their expression is also lost in ASP^cKO^P7^cKO^ tumors. Furthermore, neural stem cells have the ability to convert to a myogenic lineage and the myogenic determination factor MYOD1 can bind to neural-specific genes^46,47^. It has also been shown that *Pten* loss drives *Pax7* mRNA increases in human neural stem cells further highlighting how transcriptional regulation by PTEN may be a critical mediator of its tumor suppressor functions^48^. This may provide therapeutic vulnerability as it appears that FN-RMS cells are dependent on these lineage factors^35,49^.

Engaging the PAX7 transcriptional program, such as with DBX1, in these FN-RMS tumors by *Pten* loss highlights the necessity in understanding the complete physiological consequences of PTEN loss in tumors. This includes potential changes in the immune microenvironment as our RPPA data suggest a more immunosuppressive environment, much like other tumors with PTEN loss^50–52^. Furthermore, increasing evidence indicates that PTEN mediates its tumor-suppressive effects via not only PI3K-pathway-dependent mechanisms but also through a myriad of PI3K-pathway-independent mechanisms^11,53^. PTEN-deficient cancers are known to have synthetic essential relationships with CHD1 and PDHK1^17,18^. Concomitant *Pax7* and *Pten* loss completely rescued the deleterious effects of the latter in our FN-RMS model suggesting *Pten* loss contributes to rhabdomyosarcomagenesis in a PI3K-pathway-independent mechanism via a synthetic essential interaction. It remains unclear how pervasive these synthetic essential relationships between tumor suppressor and lineage factors are in FN-RMS or other tumors. PAX7 is only one component of the FN-RMS core-regulatory circuits (CRCs)^34,35^. Do other components of the FN-RMS CRC phenocopy this result? Furthermore, other CRCs in additional tumor types are becoming clearer; do similar mechanisms underlying genetic alteration context, cell type, and transcriptional states give rise to lineage determinants and dependencies? These are open questions that need to be empirically explored.

The combination of near-universal PTEN perturbation in human FN-RMS and PI3K-pathway-independent mechanisms in maintaining tumor identity in murine FN-RMS indicates PTEN’s role in activating RMS-specific transcriptional outputs. This work intertwines the interplay between context-dependent tumor suppressor loss and transcriptional activation can engage a synthetic essential pairing; here, the combination of PTEN loss and PAX7 activation not only maintains tumor growth and survival but maintains tumor identity. Here, we show that the ASP^cKO^P7^cKO^ tumors are no longer FN-RMS. The concomitant loss of PTEN and PAX7 gave rise to smooth-muscle-like tumors that lie along a spectrum of smooth myogenic differentiation from benign smooth-muscle-like tumors to those that appear like leiomyosarcoma. It is possible that the dual PTEN/PAX7 loss in our Smoothened-driven tumors allows the outgrowth of a precursor lesion to become more fully manifested. Furthermore, since PAX7 depletion in established tumors did not cause a shift to smooth-muscle differentiation, our data suggest that these fate decisions are made early on in tumorigenesis and that tumor lineage factors like PAX7 become more focused on tumor maintenance after these fate decisions are made. This may explain why development-related tumors, such as pediatric tumors like RMS, are highly dependent on transcriptional regulators^54^. Although their loss early on in tumorigenesis may alter the ultimate tumor presentation, clinically, these tumors may have become more addicted to these factors in tumor maintenance; this may allow for therapeutic intervention of these tumors—either through indirect mechanisms (such as with HDAC inhibition in FP-RMS) or with directly-targeted molecular glues or protein degraders as these technologies improve.

The presented work details how the synthetic essential relationship dictates tumor fate in vivo. This synthetic essential relationship between *Pten* and *Pax7* highlights the complex interplay between genetic alterations and transcriptional states that not only define tumorigenicity but even tumor origin and evolution. Taken together, this work illustrates how incredibly complex the interactions between the cell of origin, mutational spectrum, and transcriptional state are in dictating tumor lineage, and ultimately, therapeutic vulnerabilities.

## Methods

### Genetically engineered mouse models

All mouse strains are reported: *aP2-Cre*^22^, *R26-tdTomato* (#7914, The Jackson Laboratory (JAX))^55^, *SmoM2* (#5130, JAX)^56^, *Pten*^flox^ (#6440, JAX)^57^, *Pax7*^flox^ (#12653, JAX)^58^, *Cdkn2a*^*flox*^ (Nabeel Bardeesy^59^, *Trp53*^*flox*^ (#8462, JAX)^60^, and *Rb1*^*flox*^ (#01XC1, US NCI)^60^. All mice were fed and watered ad libitum with consistent access to food and water, and all mice were housed in a facility kept at ambient temperature and humidity with 12-h light/12-h dark cycles. Anterior necks lightly palpated while scruffed to feel for tumors and scored when first felt. All animal experiments were reviewed and approved by the SJCRH Institutional Animal Care and Use Committee.

### Cell lines, primary cell culture, and patient-derived xenografts (PDXs)

293T (Martine Roussel, SJCRH), RD (#CCL-136, ATCC), Rh18 (Children’s Oncology Group Cell Line Repository, Monrovia, CA, USA), SMS-CTR (Rene Galindo, UTSW Medical Center), Rh36 (Christopher Morton, SJCRH), TE441 (#CRL-7677, ATCC), Rh30 (#CRL-2261, ATCC), and Rh2, Rh6, Rh3, Rh4, Rh28, and Rh41 (Gerard Grosveld, SJCRH) cells were maintained in DMEM (#SH32043, HyClone) supplemented with 10% fetal bovine serum (Hyclone) and 1% antibiotic/antimycotic (#A5955, Sigma). SJRHB015721_X1 and SJRHB011_Y PDX-derived cell lines were harvested from SCID/Beige hindlimbs, dissociated, and plated in complete neurobasal media as described^20,24^. Briefly, tumors were digested by mechanical and enzymatic digestion with a buffered solution of collagenase II and trypsin for 1 h at 37 °C. Digested tumors were then filtered into single-cell suspensions and then plated into tissue-culture-treated plates. Primary rhabdospheres from ASP^cKO^ tumors and PDXs were dissociated similarly as above, except in the case of the primary mouse tumors the enzymatic digestion buffer contained collagenase B and dispase II; tumor or PDX cells were plated in low-attachment plates after sterile filtering into single-cell suspensions. Cell lines authenticated by short tandem-repeat profiling and monitored for mycoplasma contamination (Universal Mycoplasma Detection Kit, ATCC, #30-1012 K). Human skeletal muscle myoblasts obtained from Lonza (#CC-2580) and grown according to the manufacturer’s instructions. Mouse embryonic fibroblasts (MEFs) were harvested from E11.5 to 13.5 embryos. Wild-type MEFs immortalized by retroviral transduction with large T (genomic) antigen (#1778, Addgene, a gift from Bob Weinberg^61^). All cell lines and rhabdospheres were maintained in a humidified incubator at constant 37 °C and 5% CO_2_.

RMS PDX samples (SJRHB010468_X1, SJRHB010463_X16, SJRHB010927_X1, SJRHB010928_X1, SJRHB013757_X2, SJRHB012405_X1, SJRHB013758_X1, SJRHB013759_X1, SJRHB013758_X2, SJRHB015721_X1, SJRHB15720_X1, SJRHB011_X, SJRHB011_Y, SJRHB012_X, SJRHB012_Y, SJRHB012_Z, SJRHB013_X, and SJRHB000026_X1) were obtained from the Children’s Solid Tumor Network (CSTN)^31^. The CSTN serves as a repository for deidentified PDXs developed under the MAST (Molecular Analysis of Solid Tumors) protocol from NCT01050296^62^. NCT01050296 was approved by St. Jude Children’s Research Hospital’s Institutional Review Board. Informed consent was obtained from each trial participant prior to be enrolled in the MAST clinical trial.

### Histology and immunostaining

Fresh frozen or formalin-fixed paraffin-embedded tumor tissues were prepped for immunohistochemistry as described^20^. Briefly, paraffin-embedded tissues were rehydrated followed by antigen-retrieval (conditions listed in Supplementary Table 1). Frozen tissue sections were fixed in 4% paraformaldehyde overnight and cryoprotected in 30% sucrose, 2 mM MgCl_2_. Fixed tumor tissue was flash-frozen in liquid-nitrogen-cooled 2-methyl-butane and sectioned on a conventional cryostat. Hematoxylin & eosin (H&E) plus immunohistochemistry were performed using standard staining procedures. Antibodies are listed in Supplementary Table 2. Images captured on a Nikon Eclipse 80i upright fluorescent microscope or a Leica DMi 8 Thunder Imager inverted fluorescent microscope; images analyzed with either Nikon Elements Basic Research (v4.1.3) or the Leica Application Suite X v3.7.2 software. For PAX7 immunofluorescence, signal amplification with the TSA-Plus Cyanine 5 (#NEL745001KT, PerkinElmer) performed according to the manufacturer’s instructions. Images quantified with Nikon Elements Basic Research (v4.1.3) or FIJI’s (ImageJ, NIH) total nuclei cell counter. Specimens for transmission electron micrographs prepared and imaged as described in^41^. Briefly, tumor tissue was fixed in 2.5% glutaraldehyde, 2% paraformaldehyde, in 0.1 M sodium cacodylate buffer (pH 7.4) and post-fixed in 2% osmium tetroxide in 0.1 M cacodylate buffer with 0.15% potassium ferrocyanide. Samples were dehydrated in increasing ethanol concentration washes to propylene oxide. Samples embedded overnight at 70 °C in epoxy resin; ultrathin (80 nm) sections were imaged on a JEOL 1200EX TEM with an AMT XR111 or Tecnai TF20 TEM with an AMT XR41 camera.

### Fluorescence-activated cell sorting

ASP^cKO^ and ASP^cKO^P7^cKO^ tumors dissected and manually dissociated, digested, and stained for FACS prior to analysis with a FACS Aria Cellsorter (BD Biosciences) as described^20^. Briefly, tumors were mechanically and enzymatically digested in a buffered solution of collagenase B and dispase II for 1 h at 37 °C; tumor cells were then sterile filtered to single-cell suspensions and stained for 30 min with 1:50 anti-CD31-APC prior to flow cytometry. Antibody information is detailed in Supplementary Table 2. DAPI was used for live/dead cell marker.

### Viability assays and pharmacological agents

Cells were assessed for viability with CellTiterGlo (#G7570, Promega) or periodic image acquisition with confluence calculation (Incucyte S3, Essen BioScience). CellTiterGlo assays were performed according to the manufacturer’s protocol; luminescence was measured on a BioTek Synergy 2 and BioTek’s Gen5 1.11 software. Doxorubicin (#D-4000) and everolimus (#E-4040) were from LC Labs. GDC-0941 (#S1065), trametinib (#S2673) and MK-2206 (#S1078) were from Selleck Chemicals.

### Molecular cloning, viral transduction, and luciferase assays

Gene blocks (Integrated DNA Technologies (IDT), Coralville, IA, USA) designed for the murine *Pax7* mRNA coding sequence were cloned into the pBABE-puro retroviral vector (Hartmut Land, Jay Morgenstern, and Bob Weinberg, #1764, Addgene). Retroviruses packaged with pCL-Eco (Inder Varma, #12371, Addgene) and transfected into 293T cells. MEFs were transduced with conditioned 293T media containing viral particles after filtering (0.22 μm)^63^.

Lentiviruses encoding shRNAs (pLKO.1 or pLKO.3 G vectors, Bob Weinberg, #8453, Addgene^64^, or Christophe Benoist and Diane Mathis, #14748, Addgene) packaged and transfected into 293T cells, and adherent cells were transduced as the MEFs above^65^. For primary murine rhabdosphere transduction, virus was filtered and incubated overnight at 4°C in one-third volume of Lenti-X (#631232, Takara). Concentrated viral particles in 1–2 mL of complete neurobasal medium and plated in ultra-low-attachment plates for overnight transduction at 37 °C. For primary human PDX transduction, the filtered virus was added to pelleted PDX cells and incubated for 2.5 h at 37 °C.

MEFs infected with adenovirus-Cre recombinase (VVC-U of Iowa-1174) or GFP (VVC-U of Iowa-4) (University of Iowa Viral Vector Core) overnight (1 μg virus/1 mL medium).

The 900 bp *cis*-regulatory region immediately prior to the *Dbx1* transcription start site was synthesized (gene block, IDT) and subcloned into pGL4.25[*luc2CP/minP*] (#E8431, Promega). Synthesized *Pax7* cDNA was cloned into pCMV6 (Eric Olson). 293Ts were co-transfected with 280 ng pGL4.25-*Dbx1* promoter, 20 ng pCMV6-LacZ (as transfection control), and dose-dependent increases in *Pax7* cDNA. FuGene (#E2692, Promega) was transfection reagent. Luciferase activity was measured using the Promega Luciferase Assay System (Promega, E1501), and fluorescence activity was measured using the LacZ/Galactosidase Quantification Kit (FluoReporter, ThermoScientific, F-2905); both measurements were made on a BioTek Synergy 2. Luciferase activity was normalized to β-galactosidase activity relative to control^65^.

### Chromatin immunoprecipitation

Low-passage SJRHB015721_X1-adherent cells were grown to ~80% confluency; cells were collected in PBS by scraping. Cells were cross-linked in 1.1% formaldehyde in PBS, quenched with 125 mM glycine, and lysed in ChIP lysis buffer 3 [10 mM Tris-HCl (pH 8.0), 100 mM NaCl, 1 mM EDTA, 0.5 mM EGTA, 0.1% sodium deoxycholate, 0.5% N-lauroylsarcosine] with 1× protease inhibitors, 1 mM DTT, and 1 mM PMSF. The fixed and lysed cells were sonicated with a Covaris M220 (Covaris) sonicator with the following conditions: peak incident power—75, duty factor—15%, cycles per burst—200, temperature—7 °C for 12 min in 1 mL lysis buffer. Chromatin was incubated with pre-washed protein A/G Dynabeads (Life Technologies) and 4 μg PAX7 antibody (DSHB) overnight at 4 °C. After incubation, beads were washed and immunoprecipitates were eluted. DNA from eluates was isolated using standard phenol:chloroform extraction and resuspended in low-EDTA TE buffer. Supplementary Table 1 lists the ChIP-qPCR primers used. ChIP-qPCR signals were calculated as fold enrichment over IgG control antibody.

### Spectral karyotyping

ASP^cKO^ or ASP^WT^ tumors were analyzed for genomic alterations. Mice were intraperitoneally injected with 400 μL of 10 μg/mL colcemid and tumors were harvested 4 h post injection. Tumor cells were cultured in RPMI-1640 (supplemented with 10% fetal bovine serum and 1% penicillin/streptomycin) and harvested up to 10 days post culture using routine cytogenetic methods. A commercially prepared SKY probe from Applied Spectral Imaging (Carlsbad, CA, USA) was used as a probe for this analysis and manufacturer protocols were used for hybridization and detection steps. Two mice (*n* = 68 metaphase cells per genotype) from each genotype were analyzed.

### Immunoblotting and reverse-phase protein array (RPPA)

Immunoblotting was performed with standard procedures. Antibodies are listed in Supplementary Table 2. For RPPA, protein lysates were prepared according to protocols provided by the MD Anderson Cancer Center Functional Proteomics RPPA Core (supported by MD Anderson Cancer Center Support Grant #5 P30 CA016672-40). The RPPA core performed the array and analyzed the data. Log_2_-transformed data, median-centered, were used to generate the heatmap.

### In vivo tumorigenesis

Murine tumors dissected into ~27-mm^3^ cubes and placed into serum-free Media 199 (#11150-067, Gibco) supplemented with 1% antibiotic/antimycotic (#A5955, Sigma) prior to implantation into SCID/Beige (#250, Charles River Laboratories) or CB17 (#CB17SC, Taconic Biosciences) mice. Allografts implanted via flank incision and closed with VetBond (#14695B, 3 M).

Dissociated PDX samples were transduced with lentivirus for 2.5 h. One million PDX cells/mouse were resuspended in 100 μL matrigel (#354234, Corning) and implanted into the left hindlimb of adult SCID/Beige mice. Volumes calculated as π/6 x [(L + W)/2]3 ^63^; tumor volume reported as the difference between the injected and contralateral, uninjected limb.

### RNA isolation and gene expression analysis

RNA was isolated using a Qiagen miRNeasy Mini (#217004, Valencia, CA, USA) or Micro (#217084) (for sorted tumor populations and primary tumorsphere experiments) kit according to the manufacturer instructions. Reverse transcription was performed with Superscript III First Strand cDNA Synthesis Kit using random hexamer primers (ThermoFisher, #180800551). SYBR primers and Taqman probes used are listed in Supplementary Table 1. Data normalized to *Actb*/*ACTB* or 18S rRNA expression.

For microarray analysis changes between ASP^cKO^ and ASP^WT^ tumors, amplified cDNA was prepared using an Ovation Pico WTA System V2 (#3302, Nugen, San Carlos, CA) and analysis was performed on a Mouse 2.0 ST microarray (#902118, Affymetrix, Santa Clara, CA, USA)^20^. Data were imported into Partek Genome Suite 6.6, visualized by principal component analysis, batch corrected, and statistically tested using an unequal variance *t* test. Unannotated transcripts were filtered out and imported into STATA/MP 14.2 and *P* values adjusted for multiple comparisons by the Benjamini–Hochberg FDR method^66^. Volcano plots were also produced in STATA/MP 14.2 by plotting the log_10_ transformed *P* value from the unequal variance *t* test against the log ratio of expression.

CIBERSORT analysis for predicted differences in immune cell compartments within ASP^WT^ and ASP^cKO^ tumors was performed with the LM22 leukocyte gene signature^67^.

Gene expression changes between sh*DBX1*-transduced and shScrambled-transduced SJRHB015271_X1 cells, Clariom S human microarrays were used. In total, 125 ng RNA was processed using ThermoFisher (Affymetrix) Whole Transcript (WT) Plus assay kit (#902280). Labeled cDNA included in a hybridization mix incubated on the Clariom S human array for 16 h at 45 °C while rotating at 60 rpm. Cartridges were stained and washed on the Gene Chip FS450 fluidics station and scanned on the Gene Chip Scanner 3000 7G. Data were imported, visualized by principal component analysis, statistically analyzed, and rendered as described above.

Low-input RNA sequencing was performed for gene expression changes between flow cytometry-sorted tumor populations from the ASP^cKO^P7^cKO^ and ASP^cKO^ tumors. In all, 5 ng RNA was used in the Tecan Ovation RNA sequencing system v2 protocol as written to create SPIA (single primer isothermal amplification)-cDNA. Once purified, 500 ng of the sample was sheared (Covaris LE220 focused ultra-sonicator (96 microtube-50 AFA fiber plate, target base pair size of 300; shearing settings: Peak Incident Power (W) of 450, duty factor of 15%, cycles per burst 1000 with a 100 s treatment time)). Libraries were created with sheared cDNA and the KAPA Hyper-Prep kit (#KK8604, Roche). Four PCR cycles used for the cDNA amplification step and the protocol was followed according to the manufacturer’s instructions. UDI DNA indexes from Illumina (#20022370) were used.

Total stranded RNA sequencing data were processed by the internal AutoMapper pipeline. Raw reads were first trimmed (Trim-Galore version 0.60), mapped to mouse genome assembly (GRCm38) (STAR v2.7)^68^ and then the gene level values were quantified (RSEM v1.31) based on GENCODE annotation (vM22). Low count genes removed from analysis using a CPM cutoff corresponding to a count of ten reads and only confidently annotated (level 1 and 2 gene annotation) and protein-coding genes are used for differential expression analysis. Normalization factors were generated using the TMM method; counts then transformed using voom. Transformed counts analyzed using the lmFit and eBayes functions (R limma package version 3.42.2). The significantly up- and downregulated genes were defined by at least fold change >4 and *P* value <0.05.

For gene ontology (GO) analyses, Gene Set Enrichment Analysis (GSEA) or functional annotation clustering using the Database for Annotation, Visualization, and Integrated Discovery (DAVID, version 6.8) was used. For GSEA, the GSEA software^69^ version 4.0 was used and based on the pre-ranked option. Normalized enrichment score used for visualization. For DAVID^70^, functional annotation clustering using the GOTERM_BP_FAT selection was performed. Top ten unique GO terms visualized and represented as the −log_10_ *P* value.

### Statistics

Sample size and replicates are listed in Figure legends. Error bars reported as mean ± SEM and statistical significance determined by using two-tailed, unpaired Student’s *t* test for pairwise comparisons unless otherwise indicated. Kaplan–Meier survival analyses were performed by log-rank (Mantel–Cox) tests. *P* values below 0.05 considered significant and indicated by the following: *P < 0.05, **P < 0.01, ***P < 0.001, ****P < 0.0001. GraphPad Prism 9 was used for statistical tests. Statistical analyses of gene expression data described above.

### Reporting summary

Further information on research design is available in the Nature Research Reporting Summary linked to this article.

## Supplementary information

Supplementary Information

Description of Additional Supplementary Files

Supplementary Data 1

Supplementary Data 2

Supplementary Data 3

Reporting Summary

## Data Availability

Microarray and RNAseq data generated are deposited in the Gene Expression Omnibus (GSE166906). St. Jude ProteinPaint RNA sequencing data accessed through their open-resource page (https://pecan.stjude.cloud)^29^. RNA sequencing data from the US National Cancer Institute RMS dataset accessed from^30^. RNA sequencing and dependency data (CERES score)^28^ from the Broad Institute’s Cancer Dependency Map accessed from the 20Q3 data deposit (https://depmap.org/portal/). St. Jude and US NCI RMS core-regulatory circuit data are previously published and referenced, respectively^34,35^. Inter-species sequence conservation for the *DBX1* promoter was performed using the Evolutionary Conserved Regions database (https://ecrbrowser.dcode.org)^71^. Transcription factor binding sites found within the murine *Dbx1* promoter were mined from the JASPAR 2016 database (https://jaspar2016.genereg.net)^72^. All other relevant data supporting the key findings of this study are available within the article and its Supplementary Information files, Source Data file, or from the corresponding author, Mark E. Hatley (mark.hatley@stjude.org), upon reasonable request. A Reporting Summary for this article is available in a Supplementary Information file. Source data are provided with this paper.

## Source data

Source Data
