# Supplementary material for: Synthetic essentiality between PTEN and core dependency factor PAX7 dictates rhabdomyosarcoma identity
Source: Nat Commun. 2021 Sep 17;12:5520. doi: 10.1038/s41467-021-25829-4 (PMC8448747; doi:10.1038/s41467-021-25829-4)
Supplement: Supplementary file 1 — Supplementary Information [file 41467_2021_25829_MOESM1_ESM.pdf]

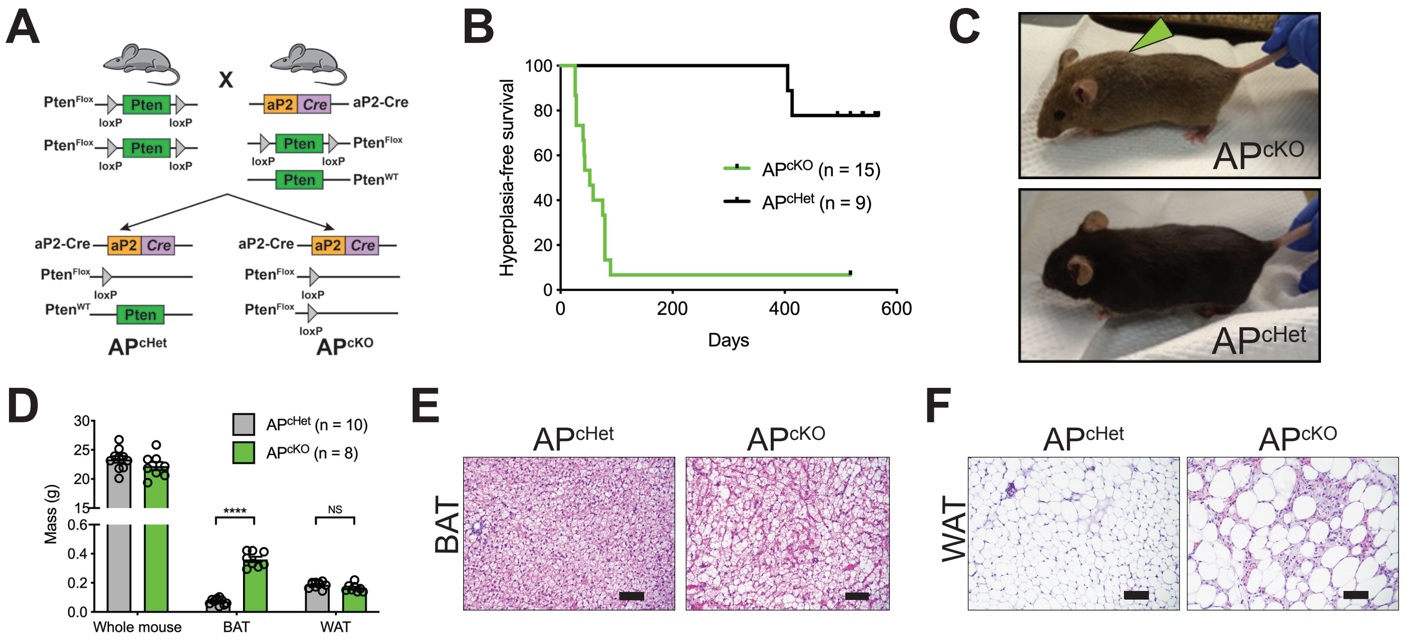

Supplementary Fig. 1. Related to Fig. 1. *Pten* loss alone in aP2-Cre expressing cells does not cause FN-RMS

(a) Breeding strategy to generate AP^{cHet} and AP^{cKO} mice.

(b) Kaplan-Meier scapular hyperproliferation-free survival curve in AP^{cKO} (green, n = 15) and AP^{cHet} (black, n = 9), Mantel-Cox log-rank $p < 0.0001$.

(c) Gross photo of AP^{cHet} and AP^{cKO} mice with enlarged scapular brown adipose tissue (BAT) in AP^{cKO} mice indicated by arrowhead.

(d) Total mouse, scapular BAT, and inguinal white adipose tissue mass two-month-old littermates from AP^{cHet} (n = 10) and AP^{cKO} (n = 8) mice (from four total litters), unpaired Student's t test, ****$p < 0.0001$ (AP^{cHet}-AP^{cKO} BAT). Data represented as mean $\pm$ SEM.

(e, f) H&E histology of scapular BAT (e) and inguinal WAT (f) from AP^{cHet} (n = 3) and AP^{cKO} (n = 3) mice. Scale bar = 50 μm.

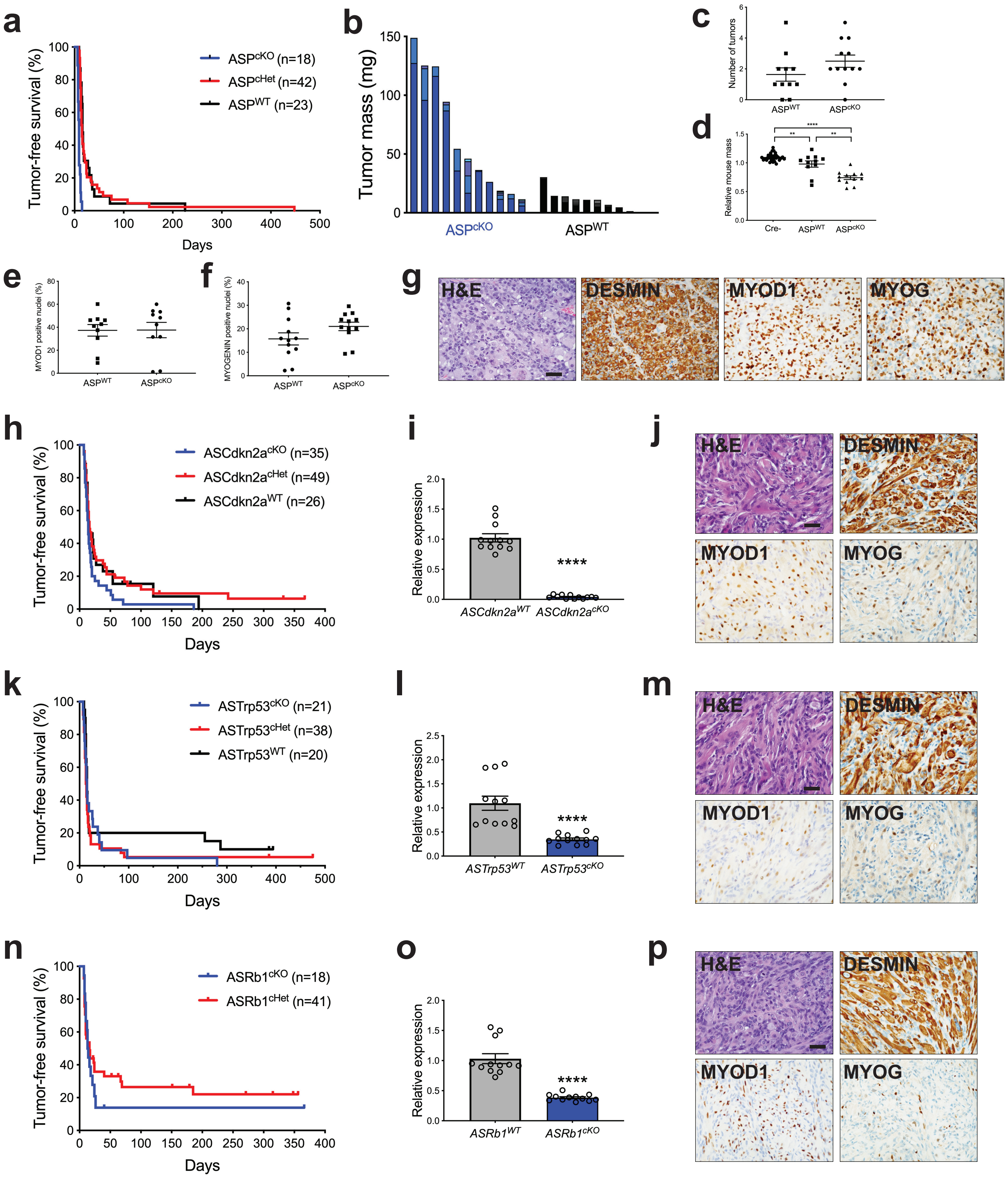

q

Tumor Suppressor	Median survival (days) WT	cKO	Log-rank (Mantel-Cox) P value	Allograft formation (tumors/total mice)
Pten	16	9	<0.0001	7/7
Trp53	15	15	0.4497	2/6
Cdkn2a	15.5	14	0.0804	0/6
Rb1	16 (cHet)	13.5	0.3846	2/8
WT				0/11

Langdon, C.G., et al., Supplementary Fig. 2

Supplementary Fig. 2. Related to Fig. 1. *Pten* accelerates and exacerbates FN-RMS development.

(a) Complete Kaplan-Meier tumor-free survival curve of the ASP^{WT} (n = 23), ASP^{cHet} (n = 42), and ASP^{cKO} (n = 18). Truncated curve at day 28 is found in Fig. 1b. Mantel-Cox log-rank $p < 0.0001$.

(b) Waterfall plot showing individual tumors dissected at P12 from ASP^{WT} (n = 11) (grayscale bars) and ASP^{cKO} (n = 12) (blue-shaded bars) mice. Cumulative masses for each individual mouse are found in Fig. 1c. Different shades within bars represent different tumors found at dissection.

(c) Quantification of total individual tumor numbers in (b) of P12 ASP^{WT} (n = 11) and ASP^{cKO} (n = 12) tumors. Difference not significant.

(d) Relative total mouse masses from ASP^{WT} (n = 11) and ASP^{cKO} (n = 12) and Cre-negative littermates. We observed during this experiment that mice from smaller litters had a higher baseline mass than mice from litters with a higher clutch size. To remedy this, we normalized the masses within littermates to compensate for the inner-litter variability in litter size. (Cre(-)-ASP^{WT} p = 0.0032, Cre(-)-ASP^{cKO} $p < 0.0001$, ASP^{WT}-ASP^{cKO} p = 0.0011).

(e, f) Percentage of MYOD1 (e) and MYOG (f) positive nuclei indexed against total number of nuclei within each field of view. Ten (e) or twelve (f) fields of view per genotype. Differences not significant.

(g) Representative histology of allografted ASP^{WT} tumor from CB17.Cg*Prkdc*^{scid}*Lyst*$^{bg\text{-}J}$/Crl mice with H&E staining and FN-RMS diagnostic IHC markers DESMIN, MYOD1, and MYOG. Scale bar = 25 μm

(h, k, n) ASCdkn2a (h), ASTrp53 (k), and ASRb1 (n) Kaplan-Maier tumor-free survival curves. Log-rank Mantel Cox p values and median survival can be found in (q).

(i, l, o) Real time PCR of *Cdkn2a* (i), *Trp53* (l), and *Rb1* (o) in ASCdkn2a, ASTrp53, and ASRb1 tumor mice, respectively, n = 4 tumors, in triplicate. Normalized to 18S rRNA and compared relative to wild-type *aP2-Cre;Smo*^{M2} tumors. All p values are < 0.0001.

(j, m, p) Representative histology and IHC staining of H&E, DESMIN, MYOD1, and MYOG in $ASCdkn2a^{cKO}$ (j), $ASTrp53^{cKO}$ (m), and $ASRb1^{cKO}$ (p) tumors. Scale bar = 25 μm. (n = 3 per genotype)

(q) Summary of AS tumors with biallelic loss of indicated tumor suppressors. Includes median survival (in days), Log-rank (Mantel-Cox) p values of Kaplan-Meier tumor-free

survival, and ratio of flank allograft formation following surgical tumor implant P values are indicated in table.

All pairwise comparisons calculated with unpaired, two-tailed, Student's t test. $^{**}p < 0.01$, $^{****}p < 0.0001$. Data represented as mean ± SEM.

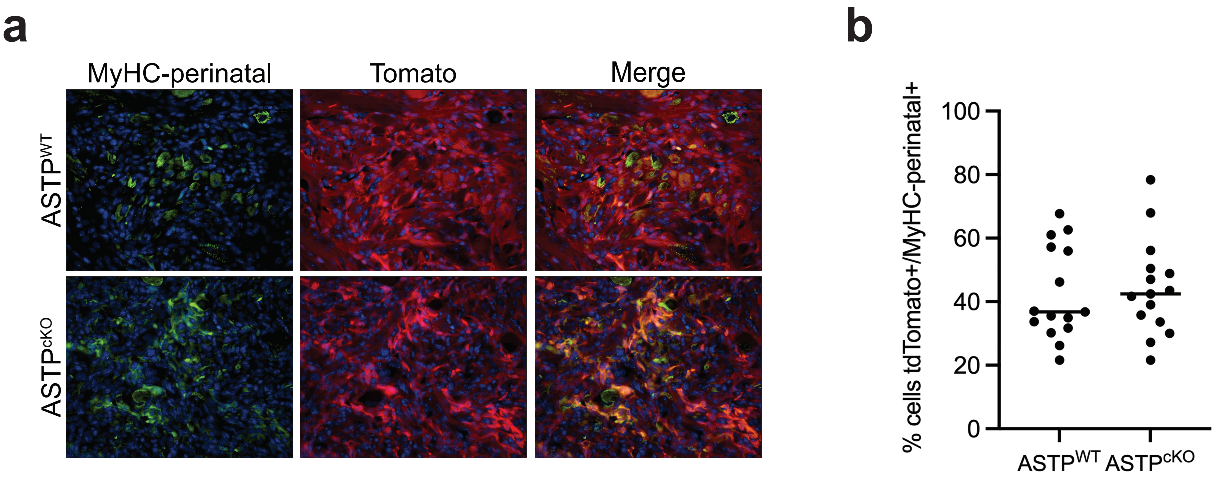

Supplementary Fig. 3, related to Fig. 2. Perinatal myosin heavy chain is not changed between ASP^{WT} and ASP^{cKO} tumors.

(a) Representative immunofluorescence from ASTP^{WT} and ASTP^{cKO} tumors stained for perinatal myosin heavy chain (MyHC-peri, green) with endogenous tdTomato fluorescence (red). DAPI nuclear stain (blue). Representative images shown, n = 3 tumors per genotype. Scale bar, 100 μm.

(b) Quantification of tdTomato+/MyHC-perinatal+ cells in tumors from (a). n = 15 fields of view per genotype. No significant difference, unpaired, two-tailed, Student's t test. Data represented as mean ± SEM.

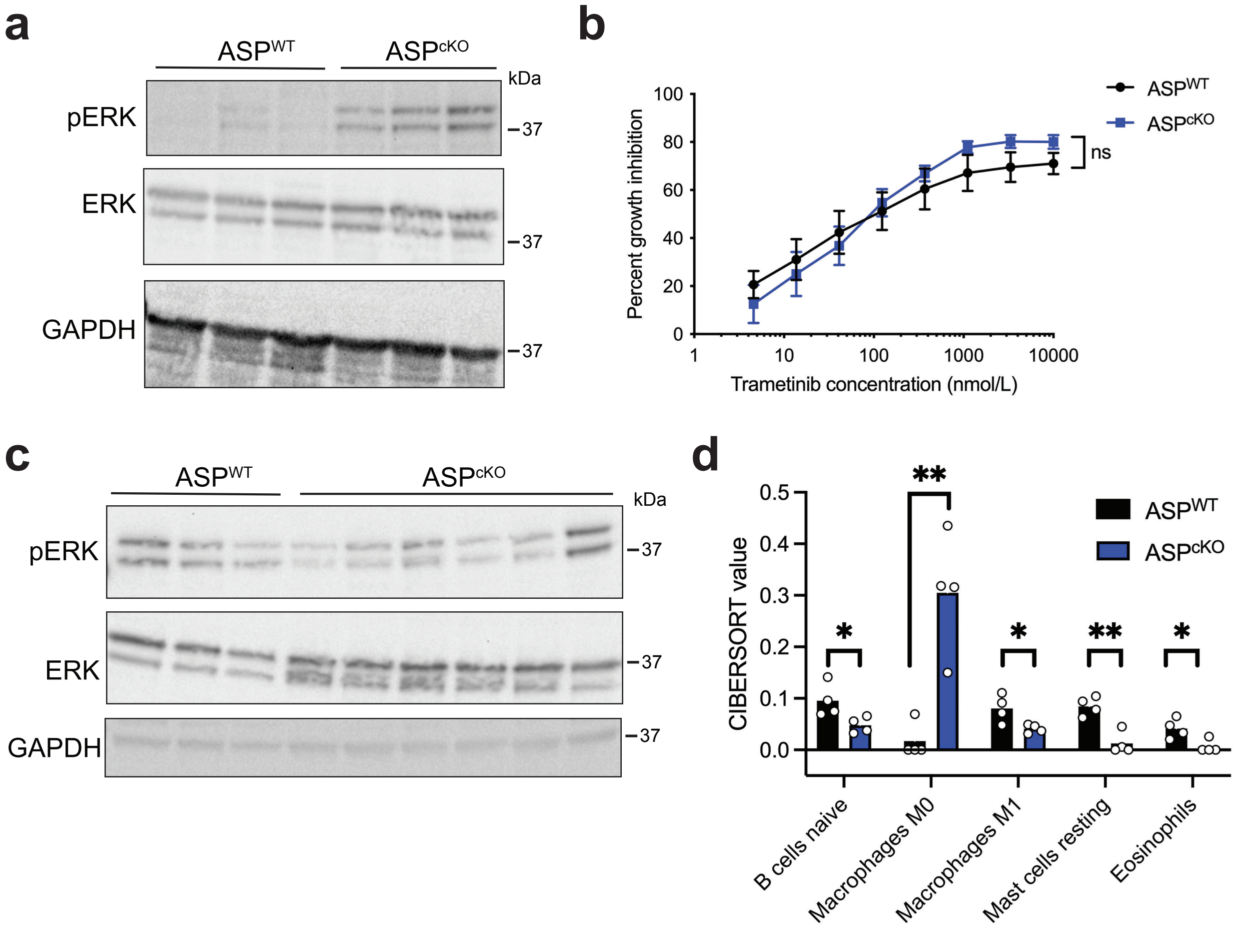

Supplementary Fig. 4, related to Fig. 3. ASP^{cKO} tumors have elevated MAPK signaling and predicted altered immunomicroenvironment compared to ASP^{WT} tumors.

(a) Immunoblots of phosphorylated ERK1/2$^{Thr202/Tyr204}$, ERK1/2, and GAPDH in ASP^{WT} and ASP^{cKO} bulk tumor lysates. n=3 per genotype.

(b) CellTiterGlo growth assays of ASP^{WT} and ASP^{cKO} primary rhabdospheres treated with trametinib. n=3 per genotype, in triplicate.

(c) Immunoblots of phosphorylated ERK1/2$^{Thr202/Tyr204}$, ERK1/2, and GAPDH in ASP^{WT} and ASP^{cKO} primary rhabdosphere cultures. n=3 for ASP^{WT} and n=6 for ASP^{cKO}.

(d) Significantly different predicted immune populations between ASP^{WT} and ASP^{cKO} tumors (n = 4 tumors per genotype) based on CIBERSORT analysis (B-cells naïve p = 0.0391, macrophages M0 p = 0.0033, macrophages M1 p = 0.0295, mast cells resting p = 0.0023, eosinophils p = 0.0237).

Unpaired, two-tailed, Student's t test. * $p < 0.05$, ** $p < 0.01$, *** $p < 0.001$. Data represented as mean $\pm$ SEM.

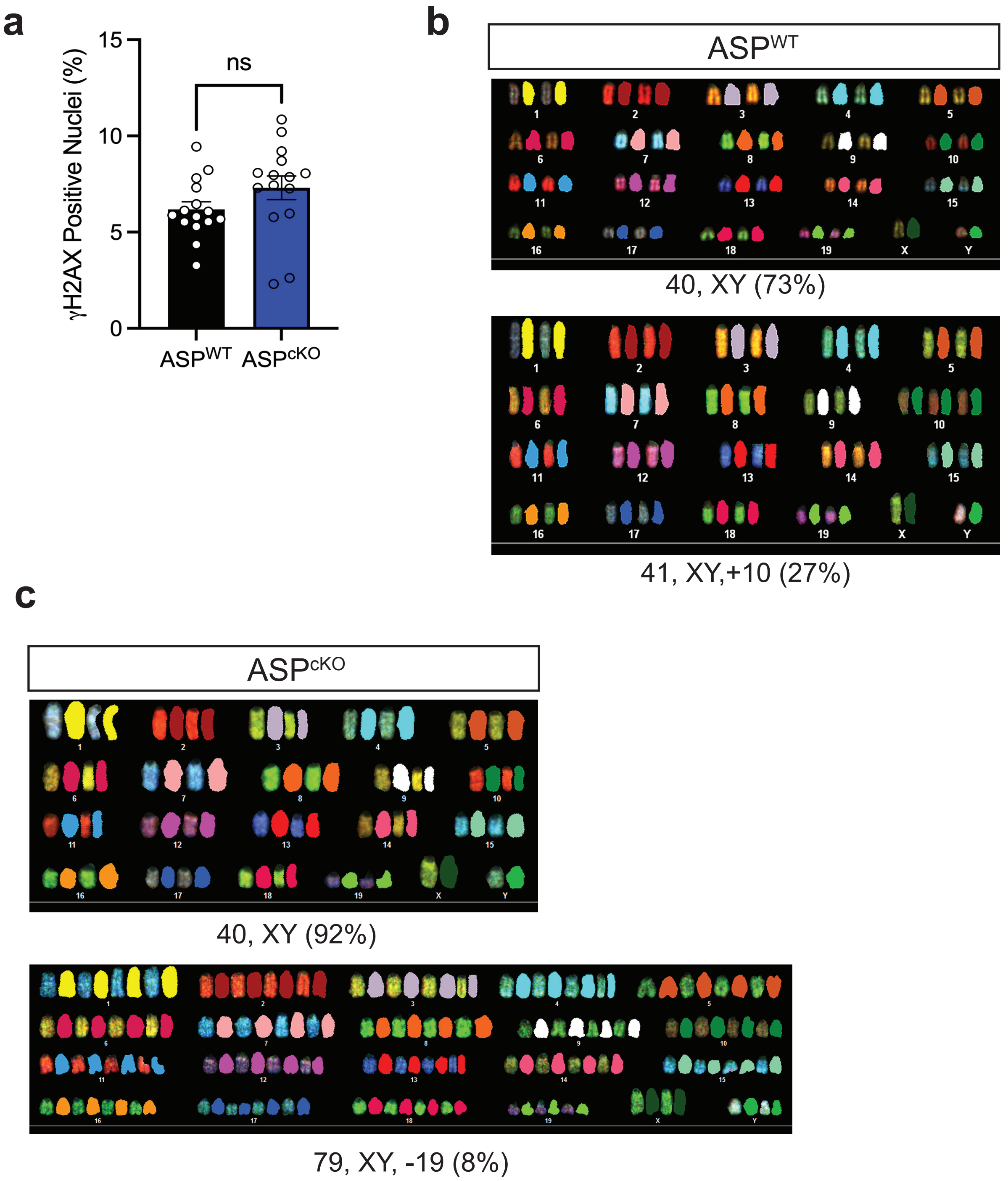
a
15
10
5
0
γH2AX Positive Nuclei (%)
ns
ASPWT
ASPcKO
b
ASPWT
40, XY (73%)
41, XY,+10 (27%)
c
ASPcKO
40, XY (92%)
79, XY, -19 (8%)

Supplementary Fig. 5, related to Fig. 3. No difference in DNA damage or extensive chromosomal alterations between ASP^{cKO} and ASP^{WT} tumors.

(a) Percent γH2.AX positive nuclei in ASP^{cKO} and ASP^{WT} tumors. n=3 tumors per genotype, 5 fields of view per tumor (15 total). Unpaired, two-tailed, Student's t test. Data represented as mean $\pm$ SEM.

(b) Representative spectral karyotyping (SKY) images are shown for ASP^{WT} tumors. Sixty-eight total metaphase cells were analyzed between two tumors. Thirty-four percent (23/68) cells exhibited chromosomal gains or losses with ten of those metaphase cells having either clonal amplification of chromosome 10 or 19. The chromosome 10 amplification is displayed in the subpanel along with a diploid, unaltered ASP^{WT} metaphase spread. Additional alterations include a clonal structural alteration within one tumor in chromosome 14 (38-41,XX,?der(14)t(?10;14)(?;?D1-3)) (9/38 cells analyzed).

(c) Representative SKY images are shown for ASP^{cKO} tumors. Sixty-eight total metaphase cells were analyzed between two tumors. Twenty-six percent of cells (18/68) exhibited non-clonal chromosomal gains or losses. Only one cell exhibited a structural chromosomal alteration between chromosomes 1 and 4 (ef (1;4), ff(1;4)). Seven percent of cells (5/68) were near tetraploid but always co-occurred with a random chromosomal alteration, usually loss. This is displayed in the subpanel at the bottom (near tetraploid with a chromosome 19 loss) along with a diploid ASP^{cKO} cell karyotype.

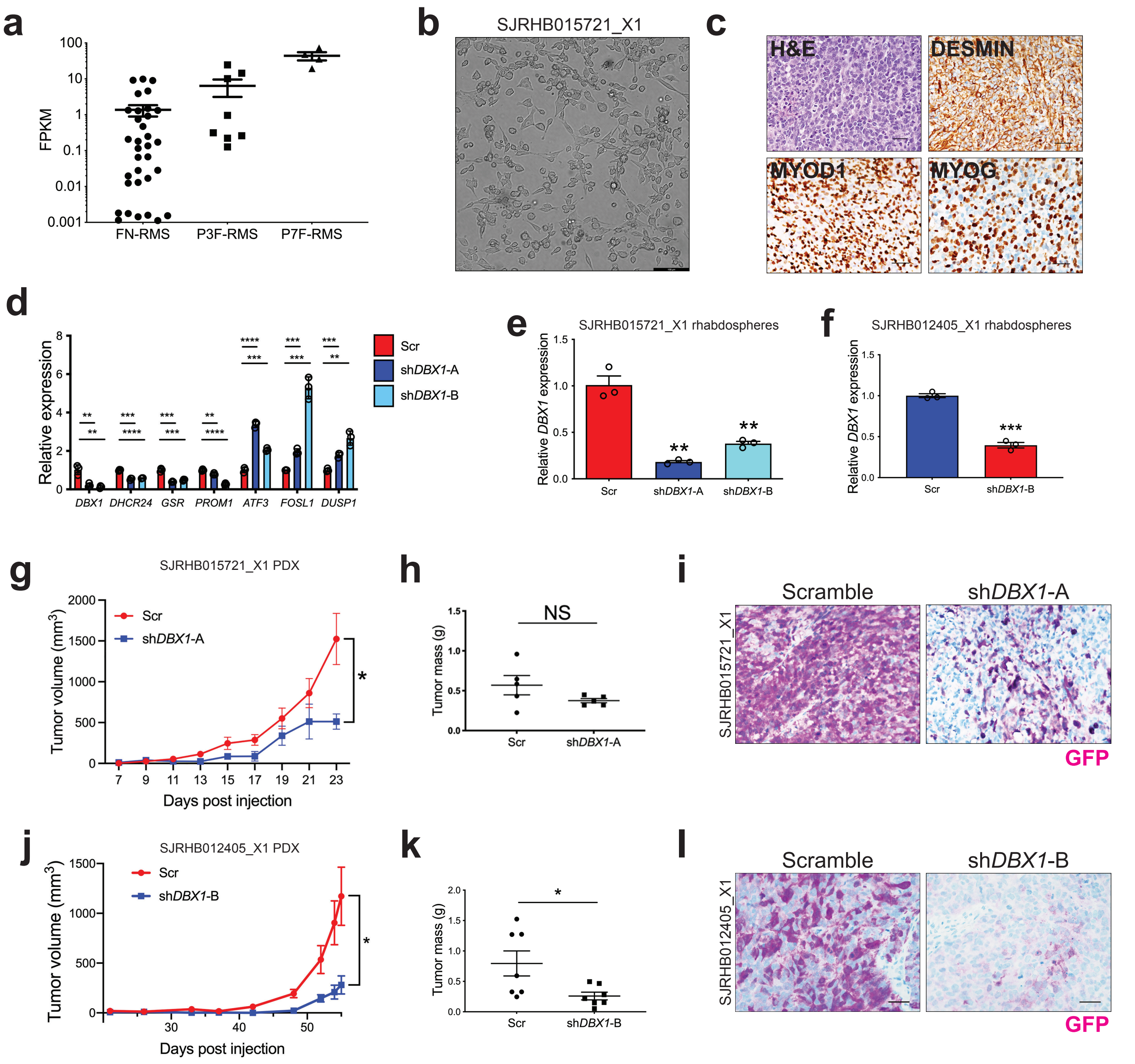

Langdon, C.G., et al., Supplementary Fig. 6

Supplementary Fig. 6. Related to Fig. 4. *DBX1* expression in human RMS tumors is critical for FN-RMS development.

(a) RNA sequencing of *DBX1* in FN-RMS (n = 33) and FP-RMS (both PAX3::FOXO1 (n = 8) and PAX7::FOXO1 (n = 4)) (as FPKM) from the St. Jude PeCan/ProteinPaint RNAseq database.

(b) Differential interference contrast (DIC) micrograph (n = 3) of PDX-derived adherent SJRHB015721_X1 cell line. SJRHB015721_X1 was originally derived from a recurrent, metastatic retroperitoneal mass from an 8-year-old male. Scale bar = 100 μm.

(c) Representative histology and H&E, DESMIN, MYOD1, and MYOG IHC from xenografted adherent SJRHB015721_X1 (n = 3) cells from the hindlimb of SCID/Beige mice. Scale bar = 25 μm.

(d) Representative real-time PCR of *DHCR24* (Scr-sh*DBX1*-A p = 0.0002, Scr-sh*DBX1*-B p < 0.0001), *GSR* (Scr-sh*DBX1*-A p = 0.0003, Scr-sh*DBX1*-B p = 0.0008), *PROM1* (Scr sh*DBX1*-A p = 0.0080, Scr-sh*DBX1*-B p < 0.0001), *ATF3* (Scr-sh*DBX1*-A p < 0.0001, Scr-sh*DBX1*-B p = 0.0002), *FOSL1* (Scr-sh*DBX1*-A p = 0.0004, Scr-sh*DBX1*-B p = 0.0002), and *DUSP1* (Scr-sh*DBX1*-A p = 0.0009, Scr-sh*DBX1*-B p = 0.0015)in *DBX1* depleted SJRHB015721_X1 cells to validate microarray found in Fig. 4g, in triplicate. Normalized to 18S rRNA and compared relative to scramble.

(e) Real-time PCR of *DBX1* to confirm *DBX1* depletion in sh*DBX1*-transduced SJRHB015721_X1 rhabdospheres cells 72 hours post-transduction, in triplicate. Normalized to 18S rRNA and compared relative to scramble. (Scr-sh*DBX1*-A p = 0.0011, Scr-sh*DBX1*-B p = 0.0034).

(f) Real-time PCR of *DBX1* in primary SJRHB012405_X1 rhabdospheres transduced concomitantly with *DBX1*-targeting or control shRNAs (p = 0.0001) and harvested after 5 days, in triplicate. Expression normalized to *ACTB* and compared relative to scramble.

(g) Tumor volume (mm^3) of SJRHB015721_X1 PDXs transduced with scrambled shRNA or sh*DBX1*-A orthotopically injected into hindlimb, n = 5 each group, p = 0.0149.

(h) Final tumor mass in grams from scrambled shRNA and sh*DBX1*-A mice after dissection from mice in (g), n = 5.

(i) GFP IHC in Scrambled- or sh*DBX1*-A-transduced SJRHB015721_X1 xenografts. Scale bar = 25 μm.

(j) Tumor volume (mm^3) of SJRHB012405_X1 PDXs transduced with scrambled shRNA and sh*DBX1*-B orthotopically injected into hindlimb, n = 7 each group, p = 0.0131.

(k) Final tumor mass in grams from scrambled shRNA and sh*DBX1*-B mice after dissection from mice in (j), $n = 7$, $p = 0.0281$.

(l) GFP IHC in Scrambled or sh*DBX1*-B-transduced SJRHB012405_X1 xenografts ($n = 7$, both conditions). Scale bar = 25 μm.

p values calculated as unpaired, two-tailed Student's t test, * < 0.05, ** $p < 0.01$, *** $p < 0.001$, **** $p < 0.0001$. Data represented as mean ± SEM.

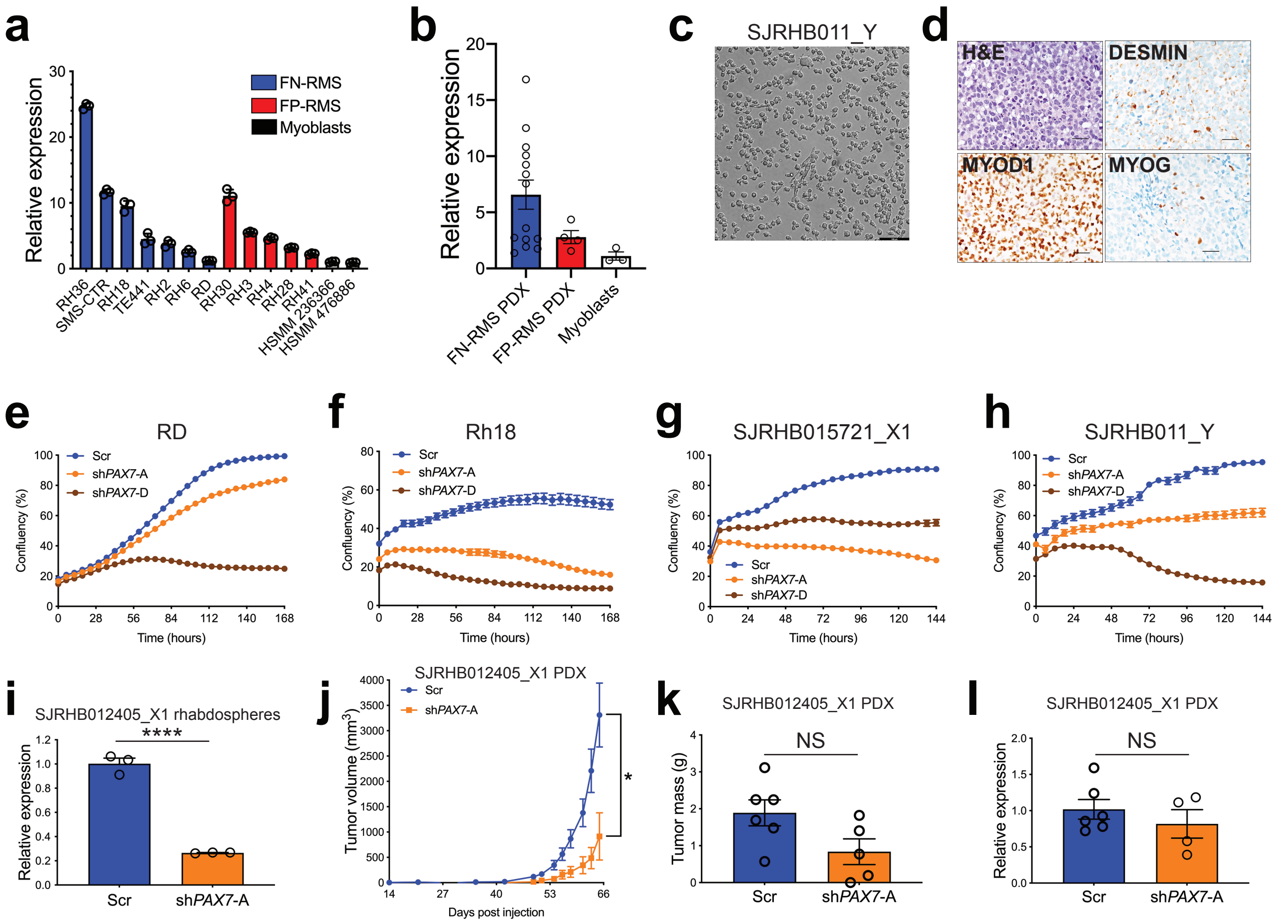

Langdon, C.G., et al., Supplementary Fig. 7

Supplementary Fig. 7. Related to Fig. 6. FN-RMS PDXs are sensitive to *PAX7* depletion.

(a) Real-time PCR of *PAX7* in FN-RMS and FP-RMS cell lines plus human skeletal muscle myoblasts. Normalized to 18S rRNA, in triplicate, and compared relative to average HSMM *PAX7* expression.

(b) Real-time PCR of *PAX7* in FN-RMS (n = 14) and FP-RMS (n = 4) PDXs plus human skeletal muscle myoblasts (n = 3), in triplicate. Normalized to 18S rRNA and compared to relative to myoblast *PAX7* expression.

(c) DIC micrograph of PDX-derived adherent SJRHB011_Y cell line (n = 3). Scale bar = 100 μm.

(d) Representative histology and H&E, DESMIN, MYOD1, and MYOG IHC from xenografted adherent SJRHB011_Y (n = 3) cells from the hindlimb of SCID/Beige mice. Scale bar = 25 μm.

(e-h) Proliferation assay represented as percent confluency following *PAX7* knock-down in RD (e), Rh18 (f), SJRHB017521_X1 (g), and SJRHB011_& (h), n = 30 (six wells, five images taken per well).

(i) Real-time PCR of *PAX7* in SJRHB012405_X1 primary rhabdospheres transduced from same pool of cells in (h), 5 days post-transduction, in triplicate, normalized to *ACTB* and compared relative to scramble, $p < 0.0001$.

(j) Tumor volume (mm^3) of SJRHB012405_X1 PDXs transduced with scrambled shRNA (n = 6) and sh*PAX7*-A (n = 5) and injected orthotopically into hindlimb, $p = 0.0164$.

(k) Final tumor mass in grams from scrambled shRNA (n = 6) and sh*DBX1*-B (n = 5) mice after dissection from mice in (j). $p = 0.0638$.

(l) Real time PCR of *PAX7* in tumors transduced with scrambled shRNA (n = 6) and sh*DBX1*-B (n = 4) isolated from (j, k). Normalized to *ACTB* expression. Note that only four tumors from the sh*PAX7*-transduced arm were analyzed as one was too small to get abundant RNA.

p values calculated as unpaired, two-tailed Student's t test, * $p < 0.05$, ** $p < 0.01$, *** $p < 0.001$, **** $p < 0.0001$. Data represented as mean ± SEM.

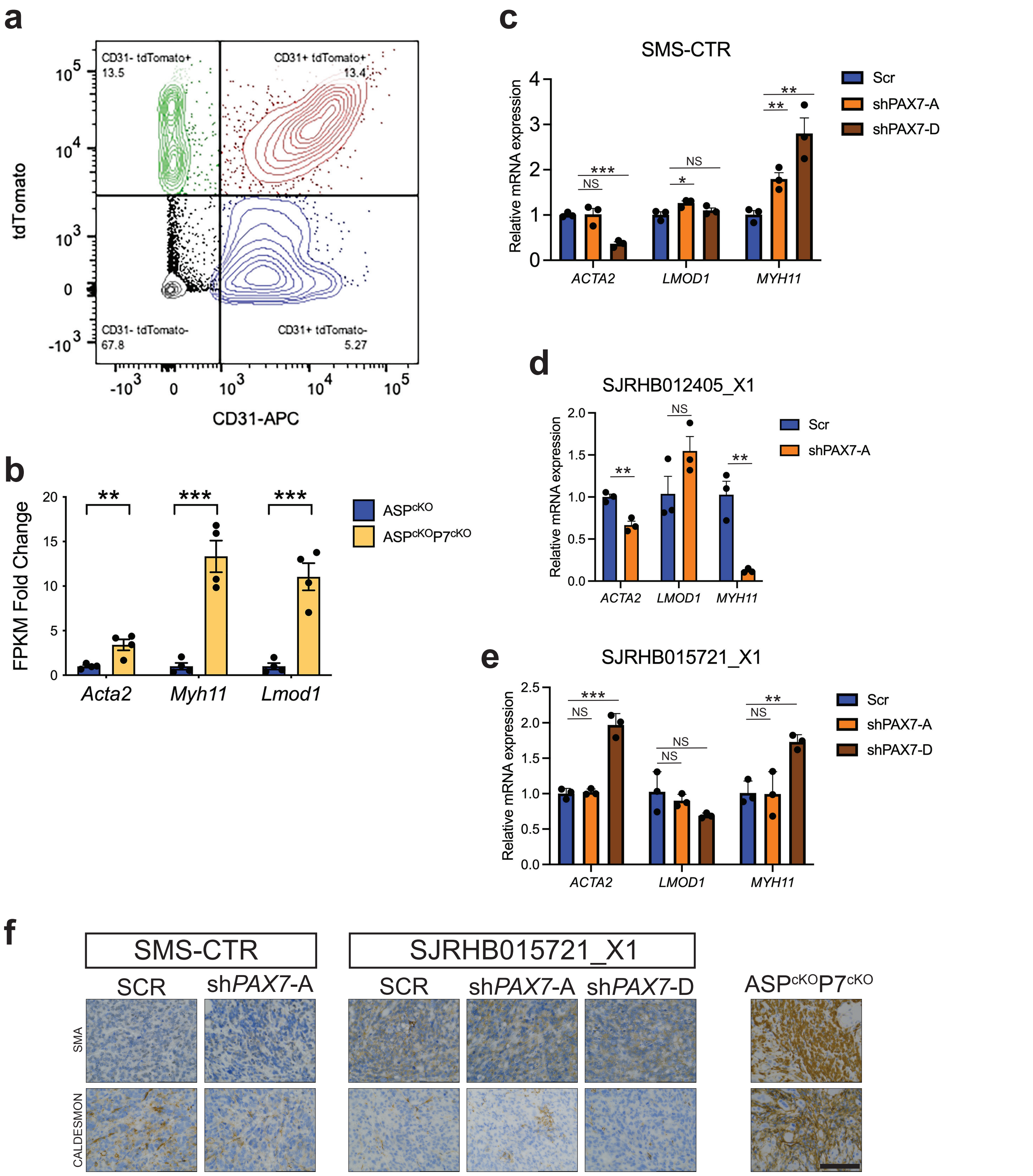

Langdon, C.G., et al., Supplementary Fig. 8

Supplementary Fig. 8, related to Fig. 8. *PAX7* depletion in human FN-RMS cells does not increase smooth muscle-related gene expression.

(a) Flow plot showing representative gating for CD31-APC(-) and tdTomato(+) tumor cells (located in the top left quadrant).

(b) FPKM fold-change of *Acta2*, *Myh11*, and *Lmod1* gene expression from the RNA-seq depicted in Fig. 8e. Presented as $ASP^{cKO}P7^{cKO}/ASP^{cKO}$ FPKM values. n=4 tumors per genotype. (*Acta2* p = 0.0086, *Myh11* p = 0.0005, *Lmod1* p = 0.0007).

(c) qPCR of *ACTA2*, *MYH11*, and *LMOD1* from SMS-CTR cells transduced with Scrambled, sh*PAX7*-A, or sh*PAX7*-D. RNA harvested 5 days post-transduction. Expression normalized to 18S. In triplicate. (*ACTA2* Scr-sh*PAX7*-D p = 0.0003, *LMOD1* Scr-sh*PAX7*-A p = 0.0341, *MYH11* Scr-sh*PAX7*-A p = 0.0092 Scr-sh*PAX7*-D p = 0.0073).

(d) qPCR of *ACTA2*, *MYH11*, and *LMOD1* from SJRHB012405_X1 cells transduced with Scrambled or sh*PAX7*-A. RNA harvested 5 days post-transduction. Expression normalized to 18S. In triplicate. (*ACTA2* p = 0.0425, *MYH11* p = 0.0485)

(e) qPCR of *ACTA2*, *MYH11*, and *LMOD1* from SJRHB015721_X1 cells transduced with Scrambled, sh*PAX7*-A and sh*PAX7*-D. RNA harvested 5 days post-transduction. Expression normalized to 18S. In triplicate. (*ACTA2* Scr-sh*PAX7*-D p = 0.0007, *MYH11* Scr-sh*PAX7*-D p = 0.0032).

(f) IHC staining for smooth muscle actin (SMA) and CALDESMON in SMS-CTR or SJRHB015721_X1 PDXs transduced with Scrambled or sh*PAX7* (n = 3, per treatment). Also shown is positive control tissue from $ASP^{cKO}P7^{cKO}$ (n = 3) tumors also stained for SMO and CALDESMON. Scale bar = 100 μm.

All p values in pairwise comparisons were determined by unpaired, two-tailed, Student's t test; * p < 0.05, **p < 0.01, ***p < 0.001. Data represented as mean $\pm$ SEM.

Supplementary Table 1. SYBR primers and Taqman probes used for qPCR or ChIP, and oligonucleotides used to generate shRNAs			
Mouse SYBR		Forward primer	Reverse primer
Actb		ATGGAGGGGAATACAGCCC	TTCTTTGCAGCTCCTTCGTT
Myf5		GACAGGGCTGTTACATTCAGG	TGAGGGAACAGGTGGAGAAC
Myod1		GTCGTAGCCATTCTGCCG	AGCACTACAGTGGCGACTCA
Myog		GTGGGAGTTGCATTCACTGG	CTACAGGCCTTGCTCAGCTC
Ckm		CAGCTTGAACTTGTTGTGGG	ACCTCCACAGCACAGACAGA
Acta1		GCCGTTGTCACACACAAGAG	CTCACTTCCTACCCTCGGC
Myh4		ACTTGGCCAGGTTGACATTG	GCAGGACTTGGTGGACAAAC
Dlx5		ACCATTCACCATCCTCACCT	AGCCCCTACCACCAGTACG
Mmp9		CTGTCGGCTGTGGTTCAGT	AGACGACATAGACGGCATCC
Foxm1		TGCCAAGATGTTGACACTCC	AGCGTTAAGCAGGAACTGGA
Bub1b		TCTGTCCCACACATCCAGAG	TGGCAAAGCAAGAGTCAGC
Ccnb1		GGCTTGGAGAGGGATTATCA	ACCAGAGGTGGAACTTGCTG
Tyms		TCAGGGTTGGTTTTGATGGT	GAGGCATTTTGGAGCAGAGT
Pvalb		CCACTTAGCTTTCAGCCACC	GAAACAAAGACGCTTCTGGC
Apod		AAGCTCGCTGGGATCTTCTC	AATTTCCATCTTGGGAAATGC
Dbx1		GGAGAAGGTCCCAGGGATAG	TAGAAGAGAAACTTCGCCCG
Pten		TCTGCAGGAAATCCCATAGC	TCGTTAGCAGAAACAAAAGGA
Pax7		GTCGGGTTCTGATTCCACAT	GCGAGAAGAAAGCCAAACAC
Trp53		TCCGACTGTGACTCCTCCAT	CTAGCATTCAGGCCCTCATC
Cdkn2a		GCAGAAGAGCTGCTACGTGA	CGTGAACATGTTGTTGAGGC
Rb1		TCTTGTCAAGTTGGCTTCCA	GAACAGATTTGTCCTTCCCG
SmoYFP		GGGATCCATTCATTCCCGCA	GCTGAACTTGTGGCCGTTTA
Gli1		GGAGACAGCATGGCTCACTA	GAGGTTGGGATGAAGAAGCA
Ptch1		AATTCTCGACTCACTCGTCCA	CTCCTCATATTTGGGGCCTT
Mouse Taqman		Vendor	Catalog #
Myh3		ThermoFisher	Mm01332463_m1
Myh8		ThermoFisher	Mm01329494_m1
Mouse shRNAs	Target sequence	Forward primer	Reverse Primer
sh*Pax7* -A	GCTGTTGATTACCTGGCCAAA	CCGGGCTGTTGATTACCTGGCCAAACTCGAGTTTGGCCAGGTAATCAACAGCTTTTTG	AATTCAAAAAGCTGTTGATTACCTGGCCAAACTCGAGTTTGGCCAGGTAATCAACAGC
sh*Pax7* -B	CCAAGATTCTGTGCCGATATC	CCGGCCAAGATTCTGTGCCGATATCCTCGAGGATATCGGCACAGAATCTTGGTTTTTG	AATTCAAAAACCAAGATTCTGTGCCGATATCCTCGAGGATATCGGCACAGAATCTTGG
Human SYBR		Forward primer	Reverse primer
PAX7		CTTCAGTGGGAGGTCAGGTT	CAAACACAGCATCGACGG
ATF3		CCTCTGCGCTGGAATCAGTC	TTCTTTCTCGTCGCCTCTTTTT
DHCR24		GCCGCTCTCGCTTATCTTCG	GTCTTGCTACCCTGCTCCTT
GSR		CACTTGCGTGAATGTTGGATG	TGGGATCACTCGTGAAGGCT
PROM1		AGTCGGAAACTGGCAGATAGC	GGTAGTGTTGTACTGGGCCAAT
FOSL1		CAGGCGGAGACTGACAAACTG	TCCTTCCGGGATTTTGCAGAT
SESN2		AAGGACTACCTGCGGTTCG	CGCCCAGAGGACATCAGTG
ACTB		GTTGTCGACGACGAGCG	GCACAGAGCCTCGCCTT
ACTA2		AAAAGACAGCTACGTGGGTGA	GCCATGTTCTATCGGGTACTTC
LMOD1		GTAAAAGGGGAGCGTAGGAAC	CTCGGGTGTTTTGGTCTTGCT
MYH11		CGCCAAGAGACTCGTCTGG	TCTTTCCCAACCGTGACCTTC
Human Taqman		Vendor	Catalog #
DBX1		ThermoFisher	hs01380082_m1
Human shRNAs	Target sequence	Forward primer	Reverse Primer
sh*PAX7* -A	GTGCAGGTCTGGTTCAGTAAC	CCGGGTGCAGGTCTGGTTCAGTAACCTCGAGGTTACTGAACCAGACCTGCACTTTTTG	AATTCAAAAAGTGCAGGTCTGGTTCAGTAACCTCGAGGTTACTGAACCAGACCTGCAC
sh*PAX7* -D	TCAGGTTTAGTGAGTTCGATT	CCGGTCAGGTTTAGTGAGTTCGATTCTCGAGAATCGAACTCACTAAACCTGATTTTTG	AATTCAAAAATCAGGTTTAGTGAGTTCGATTCTCGAGAATCGAACTCACTAAACCTGA
sh*DBX1* -A	CCTTTCATCAGATCTTCTTAT	CCGGCCTTTCATCAGATCTTCTTATCTCGAGATAAGAAGATCTGATGAAAGGTTTTTG	AATTCAAAAACCTTTCATCAGATCTTCTTATCTCGAGATAAGAAGATCTGATGAAAGG
sh*DBX1* -B	GAAGCAGAAGTACATCAGCAA	CCGGGAAGCAGAAGTACATCAGCAACTCGAGTTGCTGATGTACTTCTGCTTCTTTTTG	AATTCAAAAAGAAGCAGAAGTACATCAGCAACTCGAGTTGCTGATGTACTTCTGCTTC
Human ChIP primers		Forward primer	Reverse Primer
No PAX7 binding site control		GAACATCATGGTAGGCGCGGG	GGAAGCGAGGCGTTTACCCTC
PAX7 binding site 1		GCAGCTCCCCTGCTAAGC	ATTGCCCTACCCCCACAA
PAX7 binding site 2		CTGGTTGAGTTTGAGCGTTGG	CGGACAGCCCCTTGCAG

Supplementary Table 2. Antibodies with the applications and conditions in which they were used

Immunostaining

Antibody	Sections	Catalog/Vendor	Concentration	Antigen Retrieval	Detection or secondary antibody	Chromogen or Signal Amplification
Desmin	FFPE	RB-9014, ThermoFisher	1:500	CC1, 950-500 Roche	OmniMap rabbit, 760-4311, Roche	ChromoMap DAB, 760-159, Roche
MyoD1	FFPE	386R-18, Cell Marque	undiluted	ER2, AR9640 Leica	Bond Polymer, DS9800 Leica	ChromoMap DAB, 760-159, Roche
Myogenin	FFPE	M3559, Dako	1:200	Target Retrieval pH9, S2367 DAKO	Mouse on mouse polymer HRP MM510L, Biocare Medical	ChromoMap DAB, 760-159, Roche
Ki67	FFPE	RM-9106, ThermoFisher	1:100	CC1, 950-500 Roche	OmniMap rabbit, 760-4311, Roche	ChromoMap DAB, 760-159, Roche
MHC	Frozen	MF20, DSHB	undiluted	pH 6 Citrate buffer, C9999, Sigma	anti-mouse AlexaFluor488, A11029, ThermoFisher	N/A
Myh3	Frozen	F1.652, DSHB	undiluted	pH 6 Citrate buffer, C9999, Sigma	anti-mouse AlexaFluor488, A11029, ThermoFisher	N/A
Myh8	Frozen	N3.36, DSHB	1:10	pH 6 Citrate buffer, C9999, Sigma	anti-mouse AlexaFluor488, A11029, ThermoFisher	N/A
GFP JL8	FFPE	632381, Clontech	1:2000	CC1, 950-500 Roche	RB/MS 1:500, Abcam ab133469; OMAP rabbit, 760-4311, Roche	Discovery purple, 760-229, Roche; or ChromoMap DAB, 760-159, Roche
Smooth muscle actin	FFPE	M0851, Dako	1:30	CC1, 950-500 Roche	ARK Kit, peroxidase, K3954, DAKO	ChromoMap DAB, 760-159, Roche
Caldesmon	FFPE	04-590, Millipore	1:200	CC1, 950-500 Roche	ARK Kit, peroxidase, K3954, DAKO	ChromoMap DAB, 760-159, Roche
PTEN	FFPE	Cell Signaling, 9559	1:200	CC1, 950-500 Roche	OmniMap rabbit, 760-4311, Roche	ChromoMap DAB, 760-159, Roche
PAX7	FFPE	DSHB	1:10	pH 6 Citrate buffer, C9999, Sigma	Mouse on mouse kit, BMK-2202, Vector; anti-mouse-HRP BioRad, 170-6516	TSA-Plus-Cyanine-5, NEL745011KT, Perkin Elmer
phospho-Histone H2.AX (Ser139) (clone 20E3)	FFPE	9718, CST	1:200	CC1, 950-500 Roche	OmniMap rabbit, 760-4311, Roche	ChromoMap DAB, 760-159, Roche

Flow Cytometry

Antibody	Fluorescence	Clone	Vendor	Catalog	Concentration
Pecam1 (CD31)	APC	Mec13.3	BD Biosciences	551262	1:50

Immunoblotting

Antibody	Clone	Vendor	Catalog	Concentration	Diluent
GAPDH	6C5	Millipore	MAB374	1:10000 or 1:20000	5% milk
phospho-AKT (Ser473)	D9E	Cell Signaling	4060	1:1000	5% BSA
phospho-AKT (Thr308)	244F9	Cell Signaling	4056	1:1000	5% BSA
AKT	polyclonal	Cell Signaling	9272	1:1000	5% BSA
phospho-S6 (Ser235/Ser236)	polyclonal	Cell Signaling	2211	1:1000	5% BSA
S6	5G10	Cell Signaling	2217	1:1000	5% BSA
PAX7	polyclonal	Sigma	AV32742	1:4000	5% BSA
phospho-p70S6K (Thr389)	polyclonal	Cell Signaling	9205	1:1000	5% BSA
p70S6K	polyclonal	Cell Signaling	9202	1:1000	5% BSA
phospho-4E-BP1 (Thr37/46)	236B4	Cell Signaling	2855	1:1000	5% BSA
4E-BP1	53H11	Cell Signaling	9644	1:1000	5% BSA
PTEN	128G6	Cell Signaling	9559	1:1000	5% BSA
phospho-p44/42 MAPK (Erk1/2) (Thr202/Tyr209)	polyclonal	Cell Signaling	9101	1:1000	5% BSA
p44/42 MAPK (Erk1/2)	polyclonal	Cell Signaling	9102	1:1000	5% BSA

Chromatin Immunoprecipitation

Antibody	Vendor	Catalog	Concentration	
PAX7	DSHB	PAX7, ChIP-grade preparation	0.2 μg/μL, 4 μg total	
Normal mouse IgG	Millipore	CS200621	1 μg/μL, 4 μg total	
