# Supplementary material for: Synthetic essentiality between PTEN and core dependency factor PAX7 dictates rhabdomyosarcoma identity
Source: Nat Commun. 2021 Sep 17;12:5520. doi: 10.1038/s41467-021-25829-4 (PMC8448747; doi:10.1038/s41467-021-25829-4)
Supplement: Supplementary file 2 — Description of Additional Supplementary Files [file 41467_2021_25829_MOESM2_ESM.pdf]

Description of Additional Supplementary Files

File Name: Supplementary Data 1
Description: Differentially expressed genes (DEGs) and DAVID Functional Annotation Clustering Analysis between ASPcKO and ASPWT tumors. Related to Fig. 3.

A. Significantly ($p < 0.05$) (p values adjusted according to Benjamini-Hochberg method) differentially expressed genes (2-fold or greater) between ASPcKO and ASPWT tumors.

B. DAVID functional annotation clusters of DEGs up in ASPcKO tumors. p value derived from Fisher's exact test.

C. DAVID functional annotation clusters of DEGs down in ASPcKO tumors. p value derived from Fisher's exact test. Eleven shown as there was a repetitive GO term at the top of two clusters.

File Name: Supplementary Data 2
Description: Differentially expressed genes (DEGs) between shDBX1 and Scr-transduced SJRHB015721_X1 tumor cells. Related to Fig. 4.

A. Significantly differentially expressed genes ($p < 0.05$, with fold change larger than 1.49 or less thes 0.67-fold) between SJRHB015721_X1 cells transduced with shDBX1 or Scrambled shRNAs. shDBX1 includes only genes regulated by both shDBX1-A or shDBX1-B.

B. DAVID funcational annotation clusters of upregulated DEGs in shDBX1-transduced SJRHB015721_X1 tumor cells. p value derived from Fisher's exact test.

C. DAVID functional annotation clusters of downregulated DEGs in shDBX1-transduced SJRHB015721_X1 tumor cells. p value derived from Fisher's exact test.

File Name: Supplementary Data 3
Description: Significantly differentially expressed genes (DEGs) between ASPcKO and ASPcKOP7cKO tumor cells reveals switch from a skeletal myogenic to smooth myogenic transcriptional program. Related to Fig. 7.

A. Signifcant DEGs between ASPcKO and ASPcKOP7cKO flow cytometry-sorted tumor cells, $p < 0.05$, 4-fold differential gene expression.

B. DAVID functional annotation clustering of genes downregulated in ASPcKOP7cKO tumors. Top ten clusters shown. p value derived from Fisher's exact test.

C. DAVID functional annotation clustering of genes upregulated in ASPcKOP7cKO tumors. Top ten clusters shown. p value derived from Fisher's exact test.

D. Normalized enrichment scores of gene set enrichment analyses (GSEA) of canonical pathways. Significant gene sets ($p < 0.05$) shown. Normalized enrichment scores and p-values derived from Kolmogorov-Smirnov statistic test.
