# Supplementary material for: Synthetic essentiality between PTEN and core dependency factor PAX7 dictates rhabdomyosarcoma identity
Source: Nat Commun. 2021 Sep 17;12:5520. doi: 10.1038/s41467-021-25829-4 (PMC8448747; doi:10.1038/s41467-021-25829-4)
Supplement: Supplementary file 6 — Reporting Summary [file 41467_2021_25829_MOESM6_ESM.pdf]

nature portfolio

Corresponding author(s): Mark E. Hatley

Last updated by author(s): Aug 20, 2021

Reporting Summary

Nature Portfolio wishes to improve the reproducibility of the work that we publish. This form provides structure for consistency and transparency in reporting. For further information on Nature Portfolio policies, see our Editorial Policies and the Editorial Policy Checklist.

Statistics

For all statistical analyses, confirm that the following items are present in the figure legend, table legend, main text, or Methods section.

n/a	Confirmed	
☐	☒	The exact sample size (*n*) for each experimental group/condition, given as a discrete number and unit of measurement
☐	☒	A statement on whether measurements were taken from distinct samples or whether the same sample was measured repeatedly
☐	☒	The statistical test(s) used AND whether they are one- or two-sided<br>*Only common tests should be described solely by name; describe more complex techniques in the Methods section.*
☐	☒	A description of all covariates tested
☐	☒	A description of any assumptions or corrections, such as tests of normality and adjustment for multiple comparisons
☐	☒	A full description of the statistical parameters including central tendency (e.g. means) or other basic estimates (e.g. regression coefficient) AND variation (e.g. standard deviation) or associated estimates of uncertainty (e.g. confidence intervals)
☐	☒	For null hypothesis testing, the test statistic (e.g. *F*, *t*, *r*) with confidence intervals, effect sizes, degrees of freedom and *P* value noted<br>*Give P values as exact values whenever suitable.*
☒	☐	For Bayesian analysis, information on the choice of priors and Markov chain Monte Carlo settings
☐	☒	For hierarchical and complex designs, identification of the appropriate level for tests and full reporting of outcomes
☒	☐	Estimates of effect sizes (e.g. Cohen's *d*, Pearson's *r*), indicating how they were calculated

Our web collection on statistics for biologists contains articles on many of the points above.

Software and code

Policy information about availability of computer code

Data collection

Images captured on a Nikon Eclipse 80i upright fluorescent microscope or a Leica DMi 8 Thunder Imager inverted fluorescent microscope.

Transmission electron micrograph images were taken on a JEOL 1200EX TEM with an AMT XR111 or Tecnai TF20 TEM with an AMT XR41 camera.

Mouse tumors for RNA sequencing were dissected and manually dissociated, digested, and stained for FACS prior to analysis with a FACS Aria Cellsorter (BD Biosciences).

Cells assessed for viability with CellTiterGlo (#G7570, Promega) or periodic image acquisition with confluence calculation (Incucyte S3, Essen BioScience). CellTiterGlo assays performed according to manufacturer's protocol; luminescence measured on a BioTek Synergy 2 and BioTek's Gen5 1.11 software.

For chromatin immunoprecipitation, cells were cross-linked in 1.1% formaldehyde in PBS, quenched with 125 mM glycine, and lysed in ChIP lysis buffer 3 [10 mM Tris-HCl (pH 8.0), 100 mM NaCl, 1 mM EDTA, 0.5 mM EGTA, 0.1% sodium deoxycholate, 0.5% N-lauroylsarcosine] with 1X protease inhibitors, 1 mM DTT, and 1 mM PMSF. The fixed and lysed cells were sonicated with a Covaris M220 (Covaris) sonicator with the following conditions: peak incident power – 75, duty factor – 15%, cycles per burst – 200, temperature – 7°C for 12 minutes in 1 mL lysis buffer. Chromatin was incubated with pre-washed protein A/G Dynabeads (Life Technologies) and 4 µg PAX7 antibody (DSHB) overnight at 4° C.

RNA was isolated using a Qiagen miRNeasy Mini (#217004, Valencia, CA, USA) or Micro (#217084) (for sorted tumor populations and primary tumorsphere experiments) kit according to manufacturer instructions., Reverse transcription was performed with Superscript III First Strand cDNA Synthesis Kit using random hexamer primers (ThermoFisher, #180800551). SYBR primers or Taqman probes were used for qPCR and

ChIP-qPCR.

Data analysis

Images analyzed with either Nikon Elements Basic Research (v4.13) or the Leica THUNDER Application Suite X v3.7.2 software. Images quantified with Nikon Elements Basic Research (v4.13) or FIJI's (ImageJ, NIH) total nuclei cell counter.

Flow cytometry data analyzed with FlowJo 10.7.1.

Gene expression changes between shDBX1-transduced and shScrambled-transduced SJRHB015271_X1 cells, Clariom S human microarrays were used. 125 ng RNA was processed using Thermo Fisher (Affymetrix) Whole Transcript (WT) Plus assay kit (#902280). Labeled cDNA included in a hybridization mix incubated on the Clariom S human array for 16 hours at 45C while rotating at 60 rpm. Cartridges stained and washed on the Gene Chip FS450 fluidics station and scanned on the Gene Chip Scanner 3000 7G. Data was imported, visualized by principal component analysis, statistically analyzed, and rendered as described in Drummond et al (2018) Cancer Cell.
Low input RNA sequencing was performed for gene expression changes between flow cytometry-sorted tumor populations from the ASPcKOP7cKO and ASPcKO tumors. 5 ng RNA was used in the Tecan Ovation RNA sequencing system v2 protocol as written to create SPIA (single primer isothermal amplification)-cDNA. Once purified, 500 ng of sample was sheared (Covaris LE220 focused ultra-sonicator (96 microtube-50 AFA fiber plate, target base pair size of 300; shearing settings: Peak Incident Power (W) of 450, duty factor of 15%, cycles per burst 1000 with a 100 second treatment time)). Libraries created with sheared cDNA and the KAPA Hyper-Prep kit (#KK8604, Roche). Four PCR cycles used for the cDNA amplification step and the protocol was followed according to manufacturer's instructions. UDI DNA indexes from Illumina (#20022370) were used. For total stranded RNA sequencing, raw reads were first trimmed (Trim-Galore version 0.60), mapped to mouse genome assembly (GRCm38) (STAR v2.7) (Dobin et al (2013) Bioinformatics)) and then the gene level values were quantified (RSEM v1.3.1) based on GENCODE annotation (vM22). Low count genes removed from analysis using a CPM cutoff corresponding to a count of 10 reads and only confidently annotated (level 1 and 2 gene annotation) and protein-coding genes are used for differential expression analysis. Normalization factors generated using the TMM method; counts then transformed using voom. Transformed counts analyzed using the lmFit and eBayes functions (R limma package version 3.42.2). The significantly up- and down- regulated genes were defined by at least fold-change > 4 and p value <0.05.

For gene ontology (GO) analyses, Gene Set Enrichment Analysis (GSEA) or functional annotation clustering using the Database for Annotation, Visualization, and Integrated Discovery (DAVID, version 6.8) was used. For GSEA, the GSEA software (Subramanian et al, (2005) Proc Natl Acad Sci USA) version 4.0 was used and based on the pre-ranked option. Normalized enrichment score used for visualization. For DAVID (Huang da et al, (2009) Nat Protocol), functional annotation clustering using the "GOTERM_BP_FAT" selection was performed. Top ten unique GO terms visualized and represented as the -log10 p value.

CIBERSORT analysis for predicted differences in immune cell compartments within ASPWT and ASPcKO tumors was performed with the LM22 leukocyte gene signature.

For manuscripts utilizing custom algorithms or software that are central to the research but not yet described in published literature, software must be made available to editors and reviewers. We strongly encourage code deposition in a community repository (e.g. GitHub). See the Nature Portfolio guidelines for submitting code & software for further information.

Data

Policy information about availability of data

All manuscripts must include a data availability statement. This statement should provide the following information, where applicable:

- Accession codes, unique identifiers, or web links for publicly available datasets
- A description of any restrictions on data availability
- For clinical datasets or third party data, please ensure that the statement adheres to our policy

Microarray and RNAseq data generated are deposited in the Gene Expression Omnibus ((GSE166906) (https://www.ncbi.nlm.nih.gov/geo/query/acc.cgi?acc=GSE166906)). St. Jude ProteinPaint RNA sequencing data accessed through their open resource page (https://pecan.stjude.cloud)29. RNA sequencing data from the US National Cancer Institute RMS data set accessed from30. RNA sequencing and dependency data (CERES score)28 from the Broad Institute's Cancer Dependency Map accessed from the 20Q3 data deposit (https://depmap.org/portal/). Inter-species sequence conservation for the DBX1 promoter was performed using the Evolutionary Conserved Regions database (https://ecrbrowser.dcode.org). Transcription factor binding sites found within the Dbx1 promoter were mined from the JASPAR 2018 database (https://jaspar2018.genereg.net).

Field-specific reporting

Please select the one below that is the best fit for your research. If you are not sure, read the appropriate sections before making your selection.

☒ Life sciences ☐ Behavioural & social sciences ☐ Ecological, evolutionary & environmental sciences

For a reference copy of the document with all sections, see nature.com/documents/nr-reporting-summary-flat.pdf

Life sciences study design

All studies must disclose on these points even when the disclosure is negative.

Sample size

Post-hoc analysis of our ASP (aP2-Cre;SmoM2/+;Pten) survival curve concluded that we had 90% power when α = 0.05 (mean tumor-free survival ASPcKO (n = 18) = 9.111 days +/- 2.541 days (SD); ASPWT (n = 23) = 25.30 days +/- 23.47 days (SD). We used both male and female mice in our experiments. We did not make the same power calculation for the double conditional knockout mice or the other tumor suppressor conditional knockout mice, but targeted similar or larger sample sizes for our mouse breeding strategies.

	For non-mouse experiments, no sample size calculation was performed a priori. The sample sizes used are standards within the field and described within the Methods, the Figures, or the Figure Legends.
Data exclusions	No data were excluded.
Replication	Observations were replicated at least twice and were confirmed to be successful.
Randomization	All samples were randomly assigned.
Blinding	For in vitro and in vivo experiments, the investigators were not blinded to group allocation as they performed both the experiment and analysis; blinding was not possible.<br><br>For pathological assessment, a veterinary pathologist who did not perform the experiments was blinded to the mouse genotypes during assessment.<br><br>Only essential metadata was provided to computational biologists in order to perform analyses.

Reporting for specific materials, systems and methods

We require information from authors about some types of materials, experimental systems and methods used in many studies. Here, indicate whether each material, system or method listed is relevant to your study. If you are not sure if a list item applies to your research, read the appropriate section before selecting a response.

Materials & experimental systems

n/a	Involved in the study	
☐	☒	Antibodies
☐	☒	Eukaryotic cell lines
☒	☐	Palaeontology and archaeology
☐	☒	Animals and other organisms
☐	☒	Human research participants
☒	☐	Clinical data
☒	☐	Dual use research of concern

Methods

n/a	Involved in the study	
☒	☐	ChIP-seq
☐	☒	Flow cytometry
☒	☐	MRI-based neuroimaging

Antibodies

Antibodies used

Immunostaining
Desmin (FFPE) RB-9014, ThermoFisher 1:500
MyoD1 (FFPE) 386R-18, Cell Marque
Myogenin (FFPE) M3559, Dako 1:200
Ki67 (FFPE) RM-9106, ThermoFisher 1:100
MHC (Frozen) MF20, DSHB undiluted
Myh3 (Frozen) F1.652, DSHB undiluted
Myh8 (Frozen) N3.36, DSHB 1:10
GFP JL8 (FFPE) 632381, Clontech 1:2000
Smooth muscle actin (FFPE) M0851, Dako 1:30
Caldesmon (FFPE) 04-590, Millipore 1:200
PTEN (FFPE) Cell Signaling, 9559 1:200
PAX7 (FFPE) DSHB 1:10
phospho-Histone H2.AX (Ser139) (clone 20E3), 9718, Cell Signaling, 1:200
Flow Cytometry
Pecam1 (CD31) APC Mec13.3 BD Biosciences 551262 1:50
Immunoblotting
GAPDH 6C5 Millipore MAB374 1:10000 or 1:20000 5% milk
phospho-AKT (Ser473) D9E Cell Signaling 4060 1:1000 5% BSA
phospho-AKT (Ser308) 244F9 Cell Signaling 4056 1:1000 5% BSA
AKT polyclonal Cell Signaling 9272 1:1000 5% BSA
phospho-S6 (Ser235/Ser236) polyclonal Cell Signaling 2211 1:1000 5% BSA
S6 5G10 Cell Signaling 2217 1:1000 5% BSA
PAX7 polyclonal Sigma AV32742 1:4000 5% BSA
phospho-p70S6K (Thr389) polyclonal Cell Signaling 9205 1:1000 5% BSA
p70S6K polyclonal Cell Signaling 9202 1:1000 5% BSA
phospho-4E-BP1 (Thr37/46) 236B4 Cell Signaling 2855 1:1000 5% BSA
4E-BP1 53H11 Cell Signaling 9644 1:1000 5% BSA
PTEN 128G6 Cell Signaling 9559 1:1000 5% BSA
phospho-p44/42 MAPK (Erk1/2) (Thr202/Tyr209) polyclonal, Cell Signaling 9101, 1:1000 5% BSA
p44/42 MAPK (Erk1/2), polyclonal, Cell Signaling 9102, 1:1000 5% BSA
Chromatin Immunoprecipitation
PAX7, DSHB, Pax7 ChIP-grade preparation, 4 micrograms
Normal mouse IgG, Millipore, CS200621, 4 micrograms

Validation	Immunostaining<br>Desmin (FFPE) RB-9014, ThermoFisher - manufacturer's validation and validated by in-house Veterinary Pathology Core<br>MyoD1 (FFPE) 386R-18, Cell Marque - manufacturer's validation and validated by in-house Veterinary Pathology Core<br>Myogenin (FFPE) M3559, Dako 1:200 - manufacturer's validation and validated by in-house Veterinary Pathology Core<br>Ki67 (FFPE) RM-9106, ThermoFisher 1:100 - manufacturer's validation and validated by in-house Veterinary Pathology Core<br>MHC (Frozen) MF20, DSHB undiluted - manufacturer's validation<br>Myh3 (Frozen) F1.652, DSHB undiluted - manufacturer's validation<br>Myh8 (Frozen) N3.36, DSHB 1:10 - manufacturer's validation<br>GFP JL8 (FFPE) 632381, Clontech 1:2000 - manufacturer's validation and validated by in-house Veterinary Pathology Core<br>Smooth muscle actin (FFPE) M0851, Dako 1:30 - manufacturer's validation and validated by in-house Veterinary Pathology Core<br>Caldesmon (FFPE) 04-590, Millipore 1:200 - manufacturer's validation and validated by in-house Veterinary Pathology Core<br>PTEN (FFPE) Cell Signaling, 9559 1:200 - manufacturer's validation and validated by in-house Veterinary Pathology Core<br>PAX7 (FFPE) DSHB 1:10 - manufacturer's validation<br>phospho-Histone H2.AX (Ser139) (clone 20E3), 9718, Cell Signaling, 1:200 - manufacturer's validation and validated by in-house Veterinary Pathology Core<br>Flow Cytometry<br>Pecam1 (CD31) APC Mec13.3 BD Biosciences 551262 1:50 - manufacturer's validation<br>Immunoblotting<br>GAPDH 6C5 Millipore MAB374 1:10000 or 1:20000 5% milk - manufacturer's validation<br>phospho-AKT (Ser473) D9E Cell Signaling 4060 1:1000 5% BSA - manufacturer's validation<br>phospho-AKT (Ser308) 244F9 Cell Signaling 4056 1:1000 5% BSA - manufacturer's validation<br>AKT polyclonal Cell Signaling 9272 1:1000 5% BSA - manufacturer's validation<br>phospho-S6 (Ser235/Ser236) polyclonal Cell Signaling 2211 1:1000 5% BSA - manufacturer's validation<br>S6 5G10 Cell Signaling 2217 1:1000 5% BSA - manufacturer's validation<br>PAX7 polyclonal Sigma AV32742 1:4000 5% BSA - manufacturer's validation<br>phospho-p70S6K (Thr389) polyclonal Cell Signaling 9205 1:1000 5% BSA - manufacturer's validation<br>p70S6K polyclonal Cell Signaling 9202 1:1000 5% BSA - manufacturer's validation<br>phospho-4E-BP1 (Thr37/46) 236B4 Cell Signaling 2855 1:1000 5% BSA - manufacturer's validation<br>4E-BP1 53H11 Cell Signaling 9644 1:1000 5% BSA - manufacturer's validation<br>PTEN 128G6 Cell Signaling 9559 1:1000 5% BSA - manufacturer's validation<br>phospho-p44/42 MAPK (Erk1/2) (Thr202/Tyr209) polyclonal, Cell Signaling 9101, 1:1000 5% BSA - manufacturer's validation<br>p44/42 MAPK (Erk1/2), polyclonal, Cell Signaling 9102, 1:1000 5% BSA - manufacturer's validation<br>Chromatin Immunoprecipitation<br>PAX7, DSHB, Pax7 ChIP-grade preparation, 4 micrograms - manufacturer's validation<br>Normal mouse IgG, Millipore, CS200621, 4 micrograms - manufacturer's validation

Eukaryotic cell lines

Policy information about cell lines

Cell line source(s)	293T (Martine Roussel, SJCRH), RD (#CCL-136, ATCC), Rh18 (Children's Oncology Group Cell Line Repository, Monrovia, CA, USA), SMS-CTR (Rene Galindo, UTSW Medical Center), Rh36 (Christopher Morton, SJCRH), TE441 (#CRL-7677, ATCC), Rh30 (#CRL-2261, ATCC), and Rh2, Rh6, Rh3, Rh4, Rh28, and Rh41 (Gerard Grosveld, SJCRH) cells were maintained in DMEM (#SH32043, HyClone) supplemented with 10% fetal bovine serum (Hyclone) and 1% antibiotic/antimycotic (#A5955, Sigma). SJRHB015721_X1 and SJRHB011_Y PDX-derived cell lines were harvested from SCID/Beige hindlimbs, dissociated and plated in complete neurobasal media on low-attachment plates as described (Drummond et al (2018) Cancer Cell; Walter et al (2011) Plos One). Primary rhabdospheres from ASPcKO tumors and PDXs dissociated according to Drummond et al (2018) Cancer Cell. Human skeletal muscle myoblasts obtained from Lonza (#CC-2580) and grown according to manufacturer's instructions. Mouse embryonic fibroblasts (MEFs) were harvested from E11.5-13.5 embryos. Wild-type MEFs immortalized by retroviral transduction with large T (genomic) antigen (#1778, Addgene, a gift from Bob Weinberg (Hahn et al (2002) Mol Cell Biol)). All cell lines and rhabdospheres maintained in a humidified incubator at constant 37C and 5% CO2.
Authentication	Cell lines authenticated by short tandem repeat profiling and compared to known profiles.
Mycoplasma contamination	Cell lines routinely monitored for mycoplasma contamination (Universal Mycoplasma Detection Kit, ATCC, #30-1012K) and were all mycoplasma-negative.
Commonly misidentified lines<br>(See ICLAC register)	No misidentified lines used.

Animals and other organisms

Policy information about studies involving animals; ARRIVE guidelines recommended for reporting animal research

Laboratory animals	All mouse strains are reported: aP2-Cre (Tang et al (2008) Science), R26-tdTomato (#7914, The Jackson Laboratory (JAX)) (Madisen et al (2010) Nat Neurosci), SmoM2 (#5130, JAX) (Mao et al (2006) Cancer Res), Ptenflox (#6440, JAX) (Lesche et al (2002) Genesis), Pax7flox (#12653, JAX) (Lepper et al (2009) Nature), Cdkn2aflox (Nabeel Bardeesy, (Aguirre et al (2003) Genes Dev), Trp53flox (#8462, JAX) (Marino et al (2000) Genes Dev), and Rb1flox (#01XC1, US NCI), (Marino et al (2000) Genes Dev). Compound mutant mice (i.e. ASP = aP2-Cre;SmoM2;Pten flox) were described in Fig. 1a, Supplementary Fig. 1a, or Figure 7a. For compound AS mutant mice with either Cdkn2a flox or Trp53 flox, a similar breeding strategy to the one described in Fig. 1a was used. For ASRb1 flox, aP2-Cre;Rb1 flox/flox mice were used to breed to SmoM2/M2;Rb1 flox/+ mice. R26-tdTomato was also bred into the compound aP2-Cre mutant mice (such as those described in Fig. 2e) for tumor mouse breeding with a fluoroescent marker. Only female aP2-Cre mice were used for breeding as male aP2-Cre mice can have global Cre recombinase expression instead of being restricted to only aP2-expressing cells. For tumor generation, male and female mice were generated at Mendelian ratios with no phenotypic differences

	observed between the two sexes. For genetically engineered mouse models, the anterior necks were lightly palpated while scruffed to feel for tumors and scored when first felt.<br><br>Mouse tumor allografts implanted into SCID/Beige (#250, Charles River Laboratories) or CB17 (#CB17SC, Taconic Biosciences) mice.<br><br>All mice were fed and watered ad libitum with consistent access to food and water, and all mice were housed in a facility kept at ambient temperature and humidity with 12 hr light/12 hr dark cycles.
Wild animals	Study did not use wild animals.
Field-collected samples	No field collected samples were used.
Ethics oversight	The work reported here involving vertebrate animals was approved by the St. Jude Children's Research Hospital Institutional Animal Care and Use Committee.

Note that full information on the approval of the study protocol must also be provided in the manuscript.

Human research participants

Policy information about studies involving human research participants

Population characteristics	Seven male and seven female deidentified patient-derived xenografts (PDXs) (age range: 13 months-19 years old) were used during the study. Six PDXs were derived from samples obtained at diagnosis. Others are from recurrent patients. No treatment information is known about these samples.
Recruitment	All patients at St. Jude Children's Research Hospital are approached at intake for enrollment in the MAST (Molecular Analysis of Solid Tumor) protocol.
Ethics oversight	All patient deidentified patient-derived xenografts (PDXs) were obtained from the Childhood Solid Tumor Network (CSTN). The CSTN is the repository for PDXs developed under the MAST (Molecular Analysis of Solid Tumor) (NCT01050296) protocol that was approved by the St. Jude Children's Research Hospital Institutional Review Board.

Note that full information on the approval of the study protocol must also be provided in the manuscript.

Flow Cytometry

Plots

Confirm that:

☒ The axis labels state the marker and fluorochrome used (e.g. CD4-FITC).

☒ The axis scales are clearly visible. Include numbers along axes only for bottom left plot of group (a 'group' is an analysis of identical markers).

☒ All plots are contour plots with outliers or pseudocolor plots.

☒ A numerical value for number of cells or percentage (with statistics) is provided.

Methodology

Sample preparation	Tumors were dissected, manually dissociated, and digested for 1 hour at 37°C in 2 U/mL Collagenase B (#11088831001, Roche)/Dispase II (#04942078001, Roche), 50 mM HEPES/KOH pH 7.4; 150 mM NaCl. Following the addition of 2X volume 10% fetal bovine serum (FBS) (#SH30910.03, GE Hyclone) in phosphate buffered saline (PBS) to inactive digestion enzymes, samples were sequentially filtered through 70 µm (#22363548, Fisher) and 40 µm (#22363547, Fisher) filters to yield single cell suspensions. Single cells were blocked in 5% FBS in PBS and then stained for 30 minutes on ice with 1:50 anti-CD31-APC. Tumors endogenously expressed R26-tdTomato, so cells were sorted on tdTomato/CD31-APC status.
Instrument	FACS Aria Cell Sorter (BD Biosciences)
Software	FlowJo v10.7.1
Cell population abundance	We were only interested in the tdTomato+/CD31-APC(-) population. That ranged from 8-58% of the total tumor population.
Gating strategy	Primary gates are the "live cells" as determined by DAPI positivity from the singlet population (which was determined from the FSC/SSC gates). In the live cell gates, we were interested in the tdTomato+ cells that were CD31-APC negative.

☒ Tick this box to confirm that a figure exemplifying the gating strategy is provided in the Supplementary Information.
